# Systematics of Nothopsini (Serpentes, Dipsadidae), with a new species of *Synophis* from the Pacific Andean slopes of southwestern Ecuador

**DOI:** 10.3897/zookeys.541.6058

**Published:** 2015-12-01

**Authors:** R. Alexander Pyron, Juan M. Guayasamin, Nicolás Peñafiel, Lucas Bustamante, Alejandro Arteaga

**Affiliations:** 1 Department of Biological Sciences, The George Washington University, 2023 G St. NW, Washington, D.C. 20052 USA; 2 Centro de Investigación en Biodiversidad y Cambio Climático (BioCamb), Ingeniería en Biodiversidad y Recursos Genéticos, Facultad de Ciencias de Medio Ambiente, Universidad Tecnológica Indoamérica, Calle Machala y Sabanilla, Quito, Ecuador; 3 Tropical Herping, Av. Eloy Alfaro N39-202 y José Puerta, Ed. Montecatini, Quito, Ecuador

**Keywords:** Serpentes, Dipsadinae, Nothopsini, *Diaphorolepis*, *Synophis*

## Abstract

Within Dipsadinae, some recent authors have recognized a tribe Nothopsini containing the genera *Diaphorolepis*, *Emmochliophis*, *Nothopsis*, *Synophis*, and *Xenopholis*, on the basis of a number of putative morphological synapomorphies. However, molecular results suggest that *Nothopsis*, *Synophis*, and *Xenopholis* do not form a monophyletic group, while the remaining taxa are unsampled in recent molecular phylogenies. Here, DNA-sequence data for some *Diaphorolepis* and *Synophis* species are provided for the first time, as well as additional new sequences for *Nothopsis* and some *Synophis* species. Including these and other existing data for nothopsine species, previous studies showing that Nothopsini is not a natural group are corroborated. Nothopsini Cope, 1871 is restricted to *Nothopsis*. Diaphorolepidini Jenner, 1981 is resurrected and re-delimited to include only *Diaphorolepis*, *Emmochliophis*, and *Synophis*. Finally, *Xenopholis* remains Dipsadinae *incertae sedis*. Known material of Diaphorolepidini is reviewed to generate revised and expanded descriptions and diagnoses at the tribe, genus, and species level. Numerous cryptic species are likely present in *Synophis bicolor* and *Synophis lasallei.* Finally, a new population from the low-elevation cloud forests of SW Ecuador is reported upon, which is genetically and morphologically distinct from all other species, that is here named *Synophis zaheri* **sp. n.**

## Introduction

Within Dipsadinae (*sensu* Pyron et al. 2013), *Diaphorolepis*, *Emmochliophis*, *Nothopsis*, *Synophis*, and *Xenopholis* were historically thought to form a monophyletic group on the basis of scutellation, osteological, histological, hemipenial, and respiratory characters (see Sheil and Grant 2001). The group has been referred to as tribe Nothopsini by some authors (Savitzky 1974; Dowling and Duellman 1978). The genera *Amastridium*, *Chersodromus*, and *Ninia* have also been referred to this assemblage (Wallach 1995). Alternatively, Jenner (1981) proposed a tribe Diaphorolepidini containing *Diaphorolepis* along with *Atractus*, *Chersodromus*, *Crisantophis*, *Elapomorphus*, *Enulius*, *Gomesophis*, *Pseudotomodon*, *Ptychophis*, and *Sordellina*, while *Synophis* was placed in Philodryadini, and *Emmochliophis* was not accounted for.

Most subsequent studies have considered Nothopsini to contain only *Diaphorolepis*, *Emmochliophis*, *Nothopsis*, *Synophis*, and *Xenopholis* (see Sheil and Grant 2001; Martinez 2011). Some of these taxa, *Nothopsis* in particular, bear a strong external resemblance to Asian xenodermatids such as *Xenodermus* (Bogert 1964). In contrast, molecular phylogenetic analyses have strongly supported *Nothopsis* (Vidal et al. 2010), *Synophis* (Sheehy 2012), and *Xenopholis* (Vidal et al. 2010; Pyron et al. 2011; Grazziotin et al. 2012) as dipsadines, as does hemipenial morphology (Zaher 1999). However, these genera do not form a monophyletic group within Dipsadinae in molecular phylogenies, and are widely separated in different dipsadine clades (Vidal et al. 2010; Grazziotin et al. 2012; Sheehy 2012; Pyron et al. 2013).

Thus, the tribe Nothopsini does not appear to represent a natural group, despite the putative morphological synapomorphies uniting the taxa listed above (Savitzky 1974; Ferrarezzi 1994; Wallach 1995; Martinez 2011). Contrastingly, the strength of the molecular results suggests that these likely represent convergence, at least between *Nothopsis* and *Xenopholis*. This is not surprising, given the massive ecomorphological diversification exhibited by Dipsadinae following their adaptive radiation in the Neotropics (Cadle 1984a, b, c).

However, *Diaphorolepis* and *Emmochliophis* have still not been sampled in any molecular phylogeny, and it is thus unclear where their phylogenetic affinities lie. Morphological evidence suggests that these two genera form a clade with *Synophis* (see Hillis 1990). Furthermore, there are multiple species of *Synophis*, with potentially unclear species boundaries (Bogert 1964; Fritts and Smith 1969; Sheil 1998; Sheil and Grant 2001). Here, we report on new material from *Diaphorolepis*, *Synophis*, and *Nothopsis*, present a new molecular phylogeny, and describe a new species of *Synophis*. We review current knowledge of *Diaphorolepis*, *Emmochliophis*, and *Synophis*, and discuss species limits in these genera. Dipsadine diversity in the Andes is clearly underestimated, and new species are still being discovered in the 21st century (e.g., Salazar-Venezuela et al. 2014; Sheehy et al. 2014; Zaher et al. 2014).

## Materials

### Molecular phylogeny

Work in Ecuador was carried out under permit number MAE-DNB-CM-2015-0017. We obtained tissue samples of *Diaphorolepis wagneri* (3 specimens), *Synophis bicolor* (3), *Synophis calamitus* (1), *Synophis lasallei* (1), a new *Synophis* species (2), and *Nothopsis rugosus* (1), via fieldwork in Ecuador. The specimens are deposited at the Museo de Zoología at the Universidad Tecnológica Indoamérica (MZUTI; Tables 1, 2). We also obtained a tissue loan of the holotype of *Synophis calamitus* from Ecuador (KU 197107; Hillis 1990) from the University of Texas at Austin.

**Table 1.** Morphometric data for specimens of Diaphorolepidini species examined or from literature. Codes are: MT=maxillary teeth; IL=infralabials; SL=supralabials; PO=postoculars; V=ventrals; SC=subcaudals; D1-3=dorsal scale rows at neck, midbody, and vent; SVL=snout-vent length (mm); TL=tail length (mm). Museum codes are given in Sabaj-Perez (2013). Includes data from ReptiliaWebEcuador (Torres-Carvajal et al. 2014).

Species	Collection	MT	IL	SL	PO	V	SC	D1	D2	D3	SVL	TL	Sex
*Diaphorolepis laevis*	NMW 14860	16	10	8/9	2	157	84	19	19	17	350	145	-
*Diaphorolepis wagneri*	AMNH 49179	23	10	8	3	194	138	21	19	17	290	153	M
*Diaphorolepis wagneri*	GML 4-00014	25	10	9	2	197	133	21	19	17	355	187	F
*Diaphorolepis wagneri*	KU 75682	24	10	9	2	196	136	21	19	17	311	142	F
*Diaphorolepis wagneri*	MECN 2937	-	-	9	3	181	133	19	19	17	276	129	M
*Diaphorolepis wagneri*	MZUTI 3322	-	11	8	2	189	141	19	19	17	332	167	F
*Diaphorolepis wagneri*	MZUTI 3752	-	11	8	1	189	134	21	19	17	447	257	M
*Diaphorolepis wagneri*	MZUTI 3901	-	13	9	3	195	131	19	19	17	524	259	F
*Diaphorolepis wagneri*	NMW 18915	-	13	9	2	191	137	21	19	17	307	146	M
*Diaphorolepis wagneri*	ZSM 2708/0	25	12	9	2	193	98	21	19	17	484	200	F
*Emmochliophis fugleri*	UIMNH 78795	16	8	8	2	140	97	19	19	19	-	-	M
*Emmochliophis miops*	BMNH 1946.1.12.30	13	8	8	1	145	93	19	19	19	251	134	F
**Eastern Andes**													
*Synophis* aff. *bicolor*	FHGO 9186	-	11	9	2	164	105	19	17	17	379	184	M
*Synophis* aff. *bicolor*	MZUTI 3529	-	11	8	2	163	106	19	19	17	407	202	M
*Synophis* aff. *bicolor*	MZUTI 4180	-	11	9	2	152	100	19	19	18	457	214	M
*Synophis* aff. *bicolor*	UMMZ 91550	24/27	11	9	2	160	103	-	19	17	529	235	F
*Synophis* aff. *bicolor*	UMMZ 91551	-	-	8	2	161	105	-	19	17	535	230	F
*Synophis* aff. *bicolor*	UMMZ 91552	-	-	8	2	166	106	-	19	17	153	61	F
**Western Andes**													
*Synophis* aff. *bicolor*	BMNH 1940.2.30.31	-	-	8	2	162	118	21	19	17	408	241	M
*Synophis* aff. *bicolor*	CAS 23612	-	-	8	2	166	100	-	19	17	186	80	F
*Synophis* aff. *bicolor*	MCZ R-164530	-	11	9	2	164	116	-	19	17	367	208	-
*Synophis* aff. *bicolor*	QCAZ 10453	-	11	8	2	-	-	-	-	-	-	-	-
*Synophis* aff. *bicolor*	TCWC 66209	-	11	8	2	160	96	21	19	17	-	-	-
*Synophis* aff. *bicolor*	UMMZ 185812	-	10	8	2	165	105	-	19	17	144	66	-
*Synophis* aff. *bicolor*	UMMZ 185813	-	10	8	2	162	122	-	-	-	257	147	-
*Synophis* cf. *bicolor*	MHUA 14133	>23	12	8	2	193	127	-	19	-	-	-	M
*Synophis* cf. *bicolor*	MHUA 14577	24	11	8	2	190	131	19	19	17	-	-	-
*Synophis* cf. *bicolor*	MLS2072	-	11/10	8	2	184	127	-	19	17	407	210	M
*Synophis bicolor*	MECN 6732	-	9	8	2	174	138	19	17	17	361	236	M
*Synophis bicolor*	MECN 6733	-	9	8	2	174	132	19	19	17	406	245	M
*Synophis bicolor*	MECN 8076	-	9	8	2	183	135	19	17	17	376	233	M
*Synophis bicolor*	MZUT 257	16	9	8	2	180	136	-	19	17	-	-	-
*Synophis bicolor*	MZUTI 4175	-	11	8	2	174	143	19	19	17	365	245	M
*Synophis bicolor*	UTA R-55956	-	9	8	2	176	129	-	19	17	-	-	-
*Synophis calamitus*	KU 164208	-	9	8	1	163	125	21	19	17	142	73	-
*Synophis calamitus*	KU 197107	-	9	7	1	166	110	21	19	17	149	74	F
*Synophis calamitus*	MZUTI 3694	-	11	9	2	166	118	23	19	17	462	265	M
*Synophis calamitus*	QCAZ 11931	-	9	8	1	-	-	-	-	-	-	-	-
*Synophis lasallei*	FMNH 81313	24	-	-	2	154	112	-	21	-	292	158	F
*Synophis lasallei*	EPN S.974	-	-	-	2	156	116	-	21	-	175	90	M
*Synophis lasallei*	EPN S.975	24	-	-	2	155	119	-	21	-	354	201	M
*Synophis lasallei*	FHGO 6489	-	11	8	2	147	111	23	21	21	153	86	M
*Synophis lasallei*	FHGO 8340	-	11	8	2	153	88	21	19	17	415	199	M
*Synophis lasallei*	MCZ R-156873	-	11	7	1	147	115	-	-	-	412	206	-
*Synophis lasallei*	MECN 11250	-	10	8	2	153	98	21	19	17	412	196	F
*Synophis lasallei*	MECN 11262	-	-	8	2	154	118	21	21	17	306	145	M
*Synophis lasallei*	MECN 2220	-	10	8	2	165	117	19	19	17	294	146	M
*Synophis lasallei*	MLS/CJSP	-	-	-	2	144	101	-	-	-	300	170	M
*Synophis lasallei*	MZUTI 4181	-	11	9	2	156	29	21	21	19	272	42	M
*Synophis lasallei*	USNM 233061	-	11	9	2	156	124	-	21	-	285	160	M
*Synophis lasallei*	USNM 233062	-	11	8	2	153	126	-	22	20	360	200	-
*Synophis lasallei*	USNM 233063	-	11	8	2	151	86	23	21	19	308	197	M
*Synophis lasallei*	USNM 233064	-	11	8	2	151	-	-	21	19	270	150	-
*Synophis plectovertebralis*	UVC 11580	-	8	8	1	144	91	19	19	17	212	100	M
*Synophis plectovertebralis*	UVC 11858	-	7	7	1	147	79	19	19	17	196	76.5	F
*Synophis zaheri*	MZUTI 3353	-	8	8	2	166	112	19	19	17	351	184	M
*Synophis zaheri*	MZUTI 3355	-	9	8	2	169	111	19	19	17	372	194	M

We isolated total DNA from liver tissue or tail tips by proteinase K digestion in lysis buffer, followed by protein precipitation with guanidine thiocyanate solution and final DNA precipitation using isopropyl alcohol. We used the following pairs of primers to amplify and sequence four mitochondrial genes (12S, 16S, CYTB, ND4) and one nuclear locus (CMOS): Snake_12S_F (5’-AAACTGGGATTAGATACCCCACTAT-3’), Snake_12S_R (5’-GTRCGCTTACCWTGTTACGACT-3’), Snake_16S_F (5’-CGCCTGTTTAYCAAAAACAT-3’), and Snake_16S_R (5’-CCGGTCTGAACTCAGATCACGT-3’) from Kessing et al. (1989); Snake_Cytb_F (5’-GACCTGTGATMTGAAAACCAYCGTTGT-3’) and Snake_Cytb_R (5’-CTTTGGTTTACAAGAACAATGCTTTA-3’) from Burbrink et al. (2000); Snake_ND4_F (5’-CACCTATGACTACCAAAAGCTCATGTAGAAGC-3’) and Snake_ND4_R (5’-CATTACTTTTACTTGGATTTGCACCA-3’) from Arévalo et al. (1994); and Snake_cmosFs77 (5’-CATGGACTGGGATCAGTTATG-3’) and Snake_cmosRs78 (5’-CCTTGGGTGTGATTTTCTCACCT-3’) from Lawson et al. (2005).

We set up PCR reactions to a total volume of 25 µL containing MgCl2 2–3 mM, dNTPs 200 µM, 0.2 µM of each primer (0.8 µM in the case of ND4) and 1.25 U (16S and Cytb) or 0.625 U (ND4 and c-mos) of Taq DNA polymerase (Invitrogen). Thermocycling parameters consisted of an initial three-minute step at 94 °C; 25 to 30 cycles of 45–60 sec at 94 °C, 45 (16S and c-mos) or 60 (ND4 and Cytb) sec at 53–60 °C, 1 (16S and c-mos) or 2 (ND4 and Cytb) min at 72 °C; and a final extension of 7 min at 72 °C. We used 1.5% agarose gels to visualize the PCR products and QIAquick PCR purification Kit (QIAGEN) to remove unincorporated primers and dNTPs from every PCR reaction before they were sent to Macrogen Inc. for sequencing.

We combined these new data with the publically available sequences for *Nothopsis* and *Xenopholis* (Vidal et al. 2010; Grazziotin et al. 2012). We obtained additional sequences of *Synophis bicolor* from the Museu de Zoologia da Universidade de São Paulo (MHUA 14577 [Museo de Herpetología de la Universidad de Antioquia], from Colombia: 12S, 16S, CYTB, and CMOS) and the University of Texas, Arlington (UTA-R 55956 from Ecuador: CYTB and ND4).

We then included all publically available dipsadine species sampled for these genes. This matrix contains 24% missing data (‘-’), but these have been shown not to have deleterious effects on taxon placement and support in previous analyses (e.g., Pyron et al. 2011). Data were aligned using MAFFT (Katoh and Standley 2013) under the default parameters in Geneious 7.1.9 (Biomatters Ltd.). We determined the optimal partitioning strategy using PartitionFinder (Lanfear et al. 2012). We estimated the phylogeny using MrBayes 3.2.5 (Ronquist et al. 2012), with 4 runs of 4 chains each, run for 20 million generations with the first 25% discarded as burnin. Convergence was assumed as the average standard deviation of split frequencies went to zero and the potential scale reduction factors went to one (Ronquist et al. 2012). The GenBank accession numbers for the new and existing data are given in Appendix I.

### Morphological data

Species in *Diaphorolepis*, *Emmochliophis*, and *Synophis* have traditionally been delimited using easily determined external morphological characters (Bogert 1964; Hillis 1990). We relied here on a set of these characters, scored for museum specimens and our new material, to examine and delimit species boundaries (Table 1). For available specimens examined in person, in photographs, or in the literature, we recorded SVL and TL in mm, and counts of supralabials, infralabials, postoculars, ventrals, and subcaudals. We made cursory notes on the hemipenes of some male specimens when they were visible (Zaher 1999; Martinez 2011).

## Results

### Molecular phylogeny

**Figure 1.**
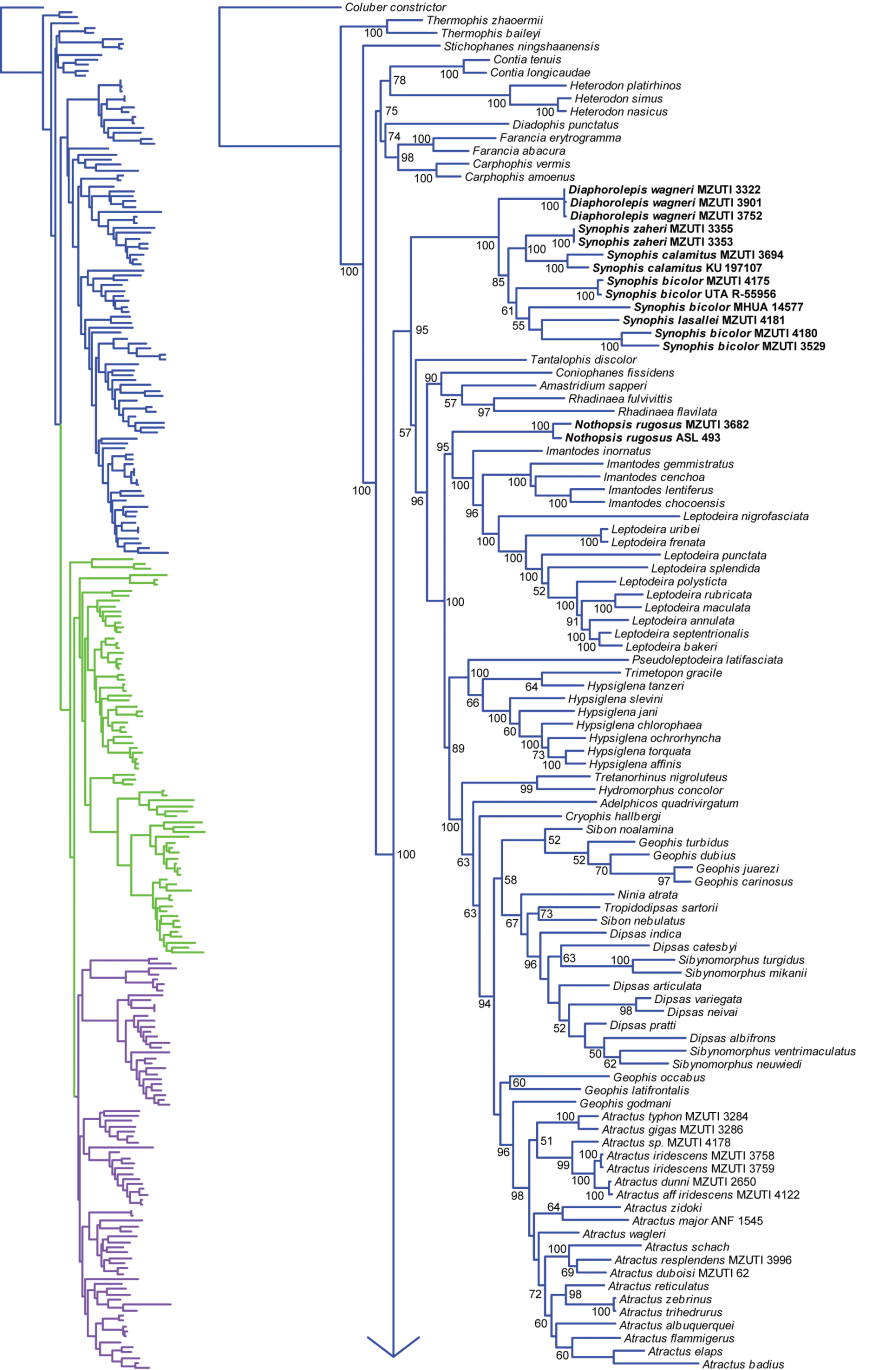
Phylogeny (part) of ~245 dipsadine species plus outgroups, based on partitioned, multi-gene Bayesian inference analysis of 3,462bp of mitochondrial and nuclear DNA. Support values given are posterior probabilities ≥50% from 15 million post-burnin generations.

**Figure 2.**
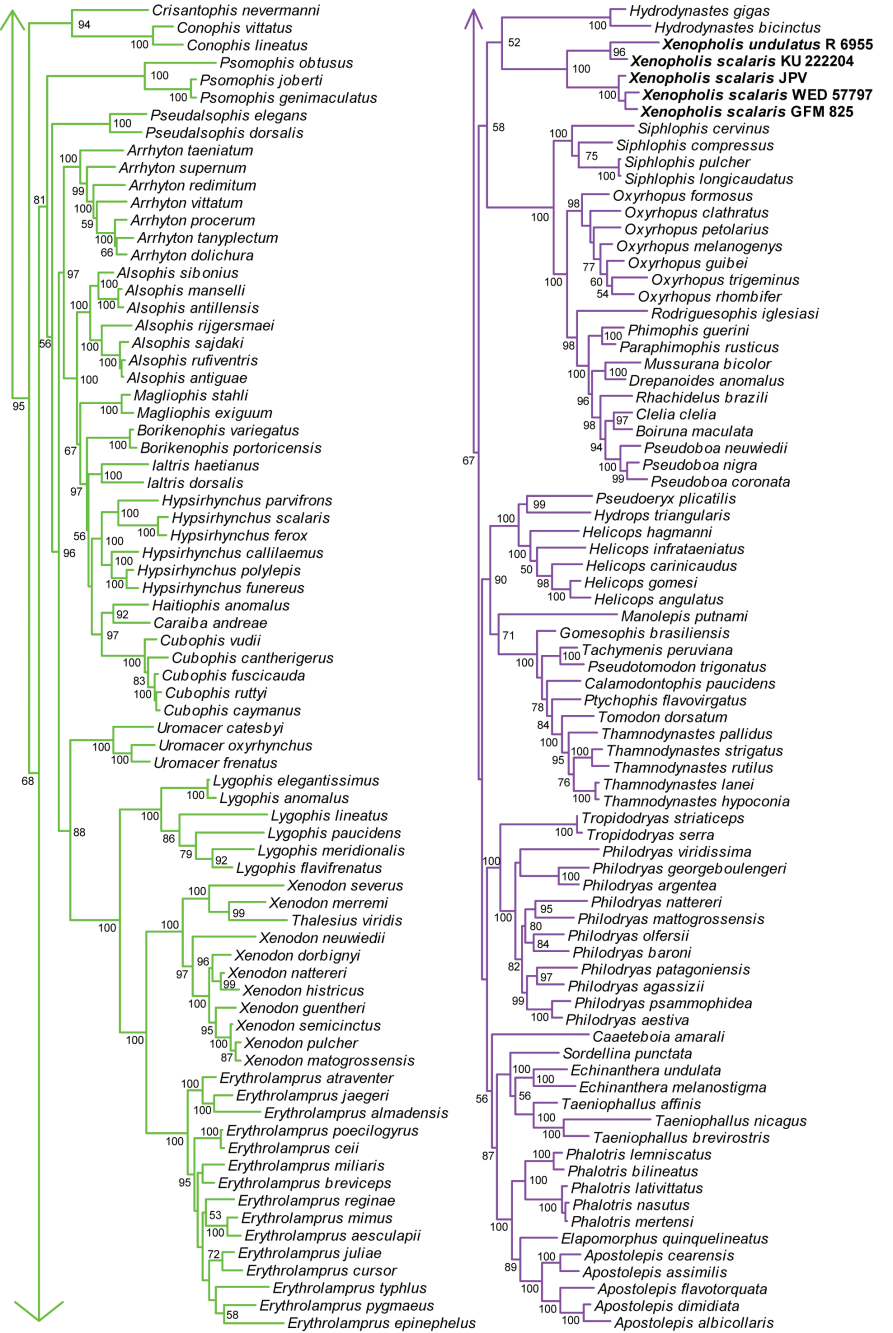
Phylogeny (part) of ~245 dipsadine species plus outgroups, based on partitioned, multi-gene Bayesian inference analysis of 3,462bp of mitochondrial and nuclear DNA. Support values given are posterior probabilities ≥50% from 15 million post-burnin generations.

The overall topology and support (Figs 1, 2) is similar to numerous recent studies (Zaher et al. 2009; Vidal et al. 2010; Pyron et al. 2011; Grazziotin et al. 2012). We consider strong support to be posterior probabilities ≥95%, following recent authors (Felsenstein 2004). Overall, there is low support for many backbone nodes, which may reflect inadequate sampling of taxa (only ~250 out of ~900 dipsadine species) or characters (only two independent loci).

Species in Dipsadinae can be broadly grouped into a primarily North American clade (*Contia* to *Carphophis* when viewing Fig. 1), a primarily Central American clade (*Diaphorolepis* to *Atractus* in Fig. 1), and a primarily South American clade (*Crisantophis* to *Apostolepis* in Fig. 2), though many species in the latter two clades range across both Central and South America. Several speciose genera in the primarily Central American clade are non-monophyletic, including *Imantodes*, *Hypsiglena*, *Geophis*, *Sibon*, *Dipsas*, *Sibynomorphus* (Fig. 1), as in previous studies (Grazziotin et al. 2012; Pyron et al. 2013).

In agreement with previous results (Grazziotin et al. 2012; Pyron et al. 2013), we find that Nothopsini is not a natural group (Fig. 1). The genus *Nothopsis* is strongly supported, and strongly placed with *Leptodeira* + *Imantodes* within the Central American clade. Correspondingly, *Xenopholis* is strongly supported and weakly nested within the South American clade, as the sister lineage to *Hydrodynastes*. It appears that one *Xenopholis scalaris* (KU 222204) from a previous study (Pyron et al. 2011) may have been misidentified, and is actually related to *Xenopholis undulatus*. This specimen is strongly supported as the sister lineage to the sampled *Xenopholis undulatus* (R-6955), to the exclusion of the three other sampled *Xenopholis scalaris*, which are strongly supported as a monophyletic group. This specimen is from the Peruvian Amazon and is pictured in Duellman and Mendelson (1995). The specimen pictured resembles the Amazonian *Xenopholis scalaris*, rather than the more xeric *Xenopholis undulatus* from the Brazilian shield. Thus, it is possible either that a curatorial or laboratory error occurred at some point, or that there is cryptic genetic diversity in *Xenopholis*.

A strongly-supported clade comprising *Diaphorolepis* and *Synophis* represents the sister to the large, primarily Central American clade that also contains *Nothopsis*. Monophyly of *Synophis* with respect to *Diaphorolepis* is weakly supported. Within a weakly paraphyletic *Synophis bicolor*, there are three deeply divergent lineages, and the sampled specimen of *Synophis lasallei.* An apparently new species of *Synophis* is the strongly-supported sister lineage of *Synophis calamitus*. The species *Synophis plectovertebralis* remains unsampled in the molecular phylogeny. Although *Emmochliophis* is not sampled, we follow previous authors in assuming a close relationship with *Diaphorolepis* and *Synophis*, given their strong resemblance (Savitzky 1974; Hillis 1990). Thus, the synapomorphies previously used to diagnose Nothopsini (Savitzky 1974; Wallach 1995) apparently represent convergence in at least three distantly related dipsadine lineages.

### Systematics

We seek here to only name clades associated Nothopsini that are strongly supported in our molecular phylogeny. Above the genus level, Nothopsini is not a natural group in any of its recent conformations. We place *Nothopsis* alone in Nothopsini Cope, 1871. We resurrect and re-delimit the tribe Diaphorolepidini Jenner, 1981 to include only *Diaphorolepis*, *Emmochliophis*, and *Synophis*. The genus *Xenopholis* is not strongly supported in any supra-generic group and remains *incertae sedis* in Dipsadinae (see Grazziotin et al. 2012).

**Figure 3.**
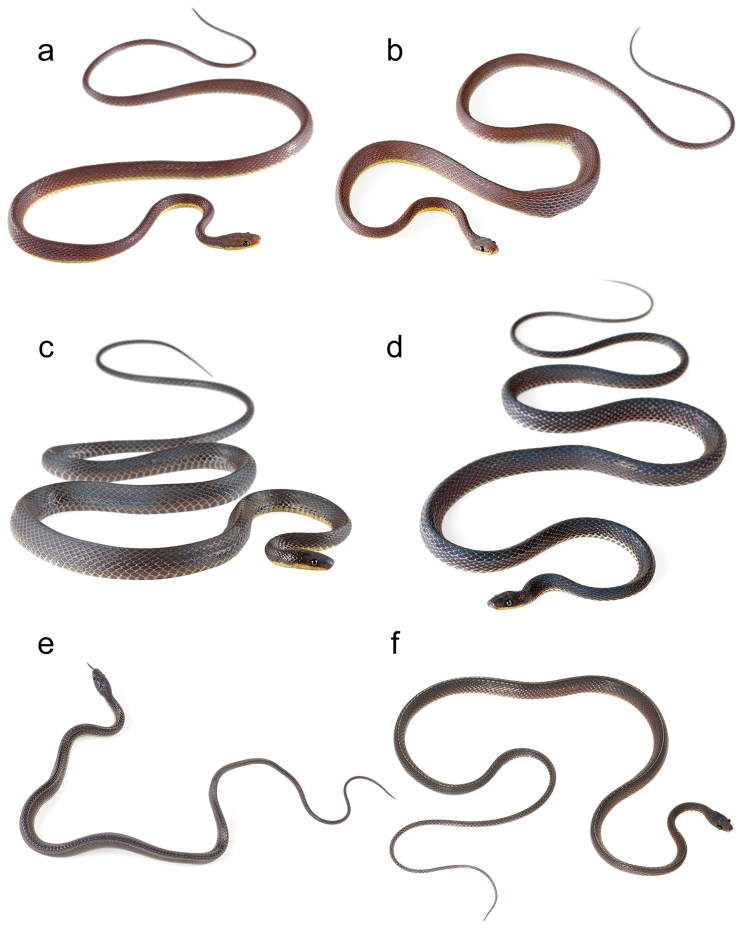
Photographs of some diaphorolepidine species in life: **a** *Synophis zaheri* MZUTI 3353 **b** *Synophis zaheri* MZUTI 3355 **c** *Synophis calamitus* MZUTI 3694 **d** *Synophis* aff. *bicolor* MZUTI 3529 **e** *Synophis lasallei* uncat., and **f** *Diaphorolepis wagneri* MZUTI 3901.

**Figure 4.**
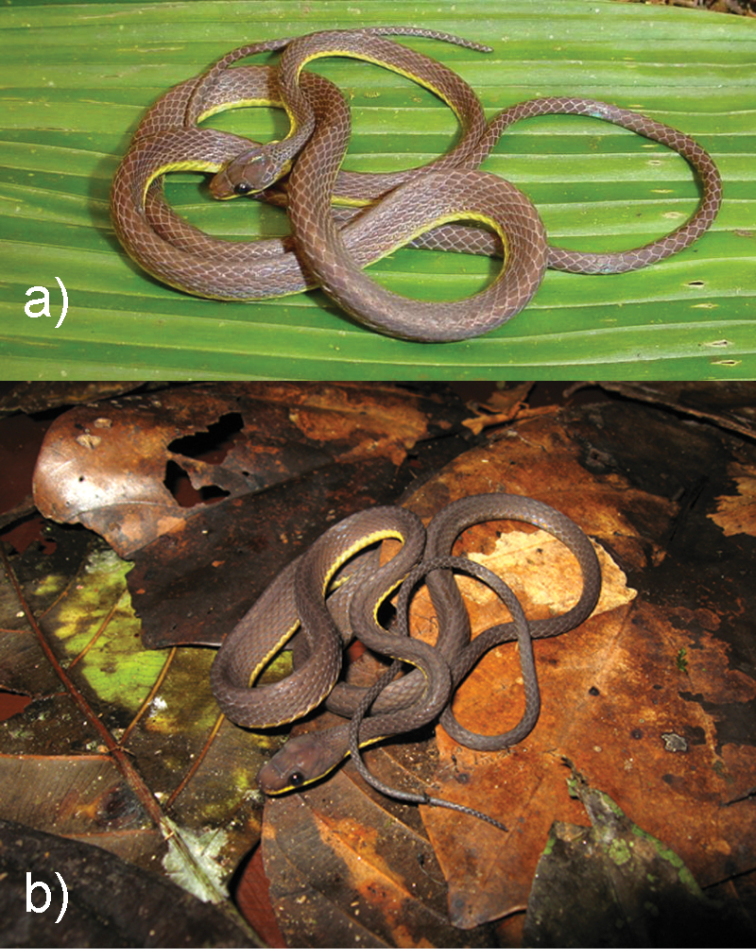
Photographs of some diaphorolepidine species in life: *Synophis bicolor* UTA R-55956 (**a**), and *Synophis* cf. *bicolor* MHUA 14577 (**b**).

**Figure 5.**
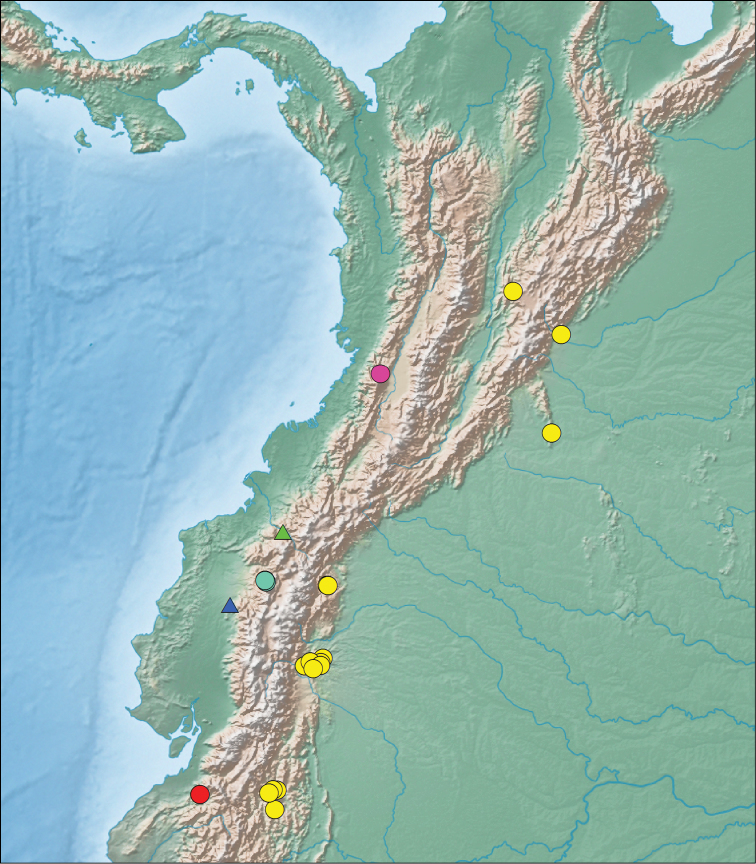
Map of vouchered localities for *Synophis lasallei* (yellow circles), *Synophis plectovertebralis* (pink circles), *Synophis calamitus* (teal circles), *Synophis zaheri* (red circles), *Emmochliophis miops* (green triangle) and *Emmochliophis fugleri* (blue triangle).

**Figure 6.**
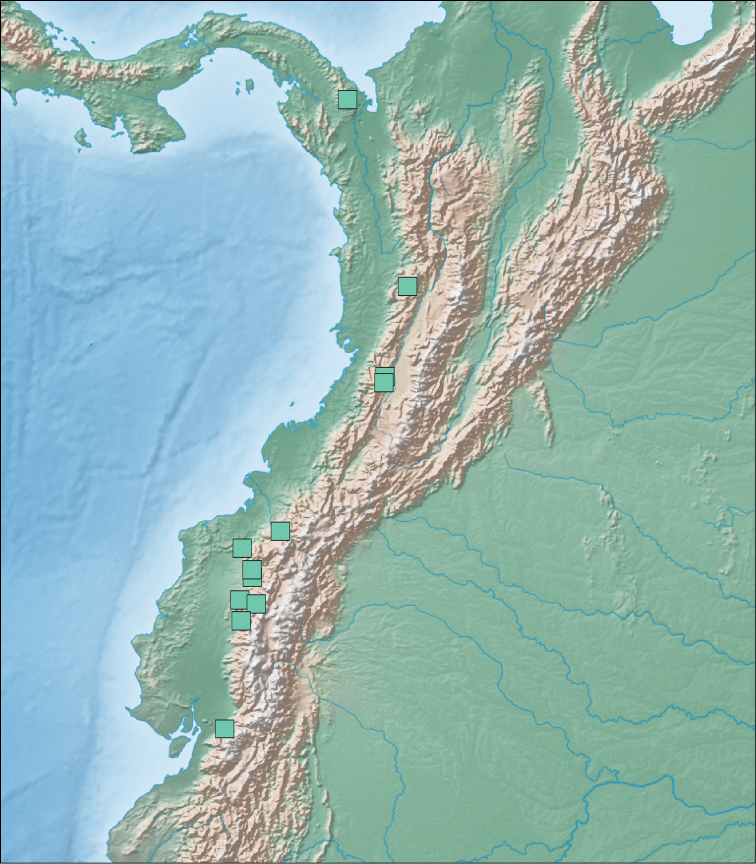
Map of vouchered localities for *Diaphorolepis wagneri* (teal squares).

**Figure 7.**
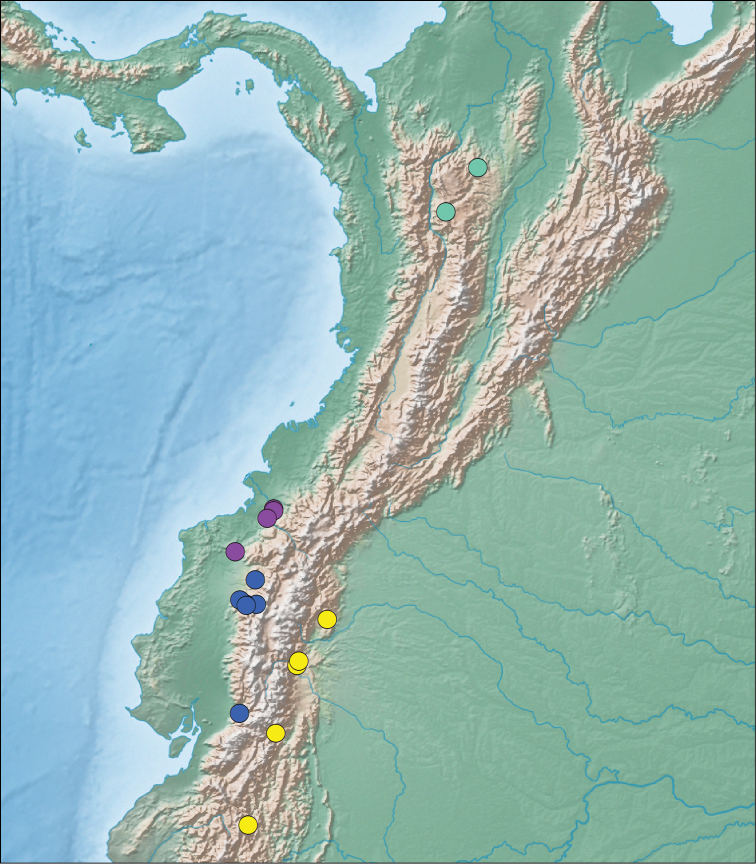
Map of vouchered localities for *Synophis bicolor* populations: *Synophis bicolor* *sensu stricto* (purple circles), western *Synophis* aff. *bicolor* (blue circles), eastern *Synophis* aff. *bicolor* (yellow circles), and *Synophis* cf. *bicolor* (teal circles).

**Figure 8.**
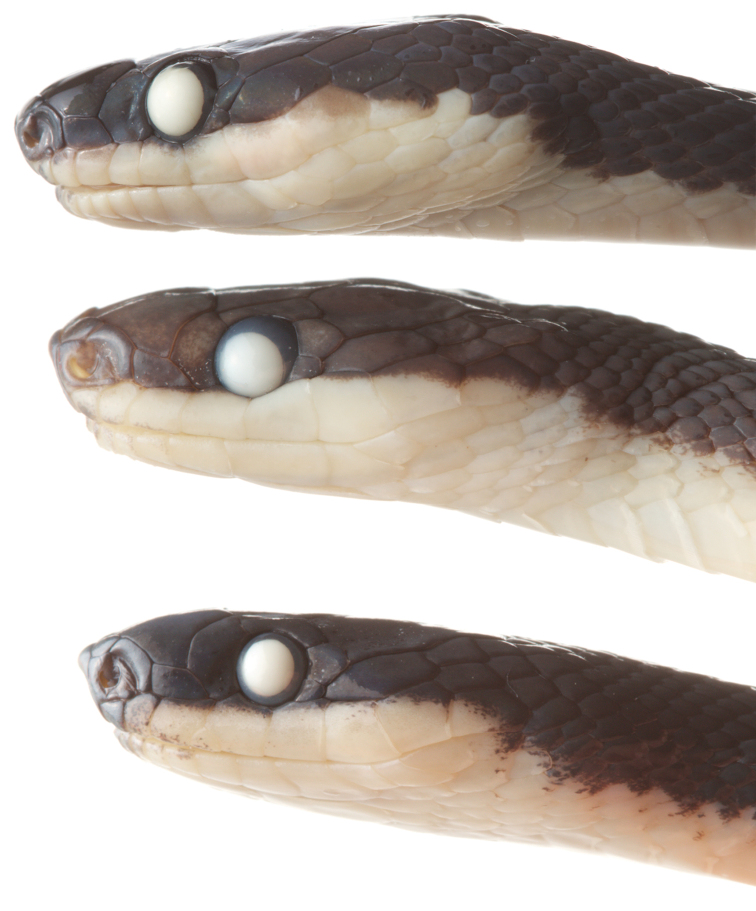
Photographs in preservation of some diaphorolepidine species. Upper: *Diaphorolepis wagneri* MZUTI 3901, Center: *Synophis zaheri* MZUTI 3355, Lower: *Synophis calamitus* MZUTI 3694.

**Figure 9.**
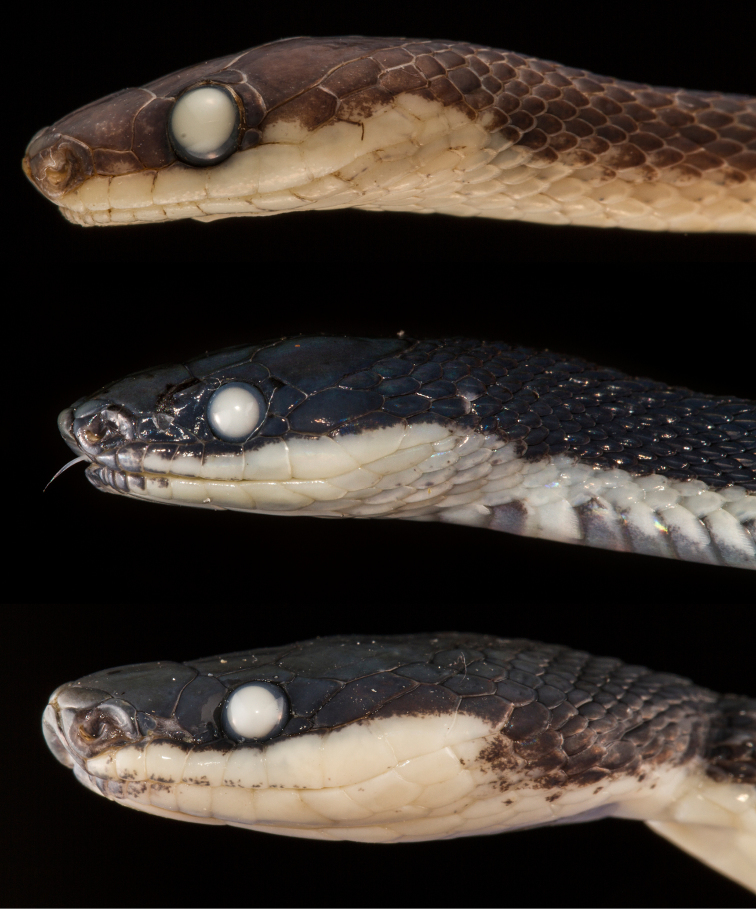
Photographs in preservation of some diaphorolepidine species. Upper: *Synophis bicolor* MZUTI 4175, Middle: *Synophis lasallei* MZUTI 4181, Lower: *Synophis* aff. *bicolor* MZUTI 4180.

Our molecular and morphological data (Tables 1–3; Figs 1, 2) also corroborate previous authors in finding that genus and species boundaries within Diaphorolepidini are unclear and in need of revision (Sheil and Grant 2001). We here provide photographs and range maps of representative material (Figs 3–9). A number of issues are immediately apparent, and can be addressed with our results. We outline these below.

**Table 2.** Vouchered localities for specimens of Diaphorolepidini species examined or from literature. In general, localities are given verbatim as transcribed from the literature, museum records, or field notes. Co-ordinates represent georeferencing attempts from gazetteers under standard guidelines, though some variation from the exact collecting locality will inevitably be present. Similarly, elevations are taken from Google Earth, and may not exactly match the elevations as originally reported. Museum codes are given in Sabaj-Perez (2013). Includes data from ReptiliaWebEcuador (Torres-Carvajal et al. 2014).

Species	Collection Number	Locality	Latitude	Longitude	Elev.
*Diaphorolepis wagneri*	GML 4-00014	Panama Darien, Cerro Mali, in Serrania del Darien	8.128557	-77.253498	1268
*Diaphorolepis wagneri*	MECN 2937	Canandé, Ecuador	0.529930	-79.035410	596
*Diaphorolepis wagneri*	MZUTI 3322	Milpe, Ecuador	0.034890	-78.867130	1076
*Diaphorolepis wagneri*	MZUTI 3901	Mashpi Lodge, Ecuador	0.164030	-78.870730	1068
*Diaphorolepis wagneri*	NMW 18915	El Palmar, Canar, Ecuador	-2.533300	-79.333300	325
*Diaphorolepis wagneri*	QCAZ 380	Ecuador, Cotopaxi, Las Pampas	-0.348360	-79.076010	1238
*Diaphorolepis wagneri*	QCAZ 381	Ecuador, Pichincha, Tandapi	-0.415220	-78.797280	1457
*Diaphorolepis wagneri*	QCAZ 8450	Ecuador, Cotopaxi, Pucayacu–Sigchos	-0.702730	-79.056810	974
*Diaphorolepis wagneri*	QCAZ 8782	Imbabura Lita, Ecuador	0.815270	-78.388350	865
*Diaphorolepis wagneri*	UVC 12187	18km East of San Jose de Palmar, Colombia	4.966667	-76.233333	1546
*Diaphorolepis wagneri*	UVC 5254	Colombia, Cali, Pichinde, Farallones de Cali	3.433400	-76.616680	1614
*Diaphorolepis wagneri*	UVC 5255	Colombia, Pance, Camino a Corea, Pance, Farallones de Cali	3.328340	-76.638650	1632
*Emmochliophis fugleri*	UIMNH 78795	4 km. E Río Baba Bridge, 24 km. S Santo Domingo de los Colorados, Pichincha, Ecuador	-0.435562	-79.246212	618
*Emmochliophis miops*	BMNH 1946.1.12.30	Parambas (Imbabura), Ecuador	0.805000	-78.350833	1105
**Eastern Andes**					
*Synophis* aff. *bicolor*	FHGO 9186	Río Zopladora, Ecuador	-2.611510	-78.472174	1677
*Synophis* aff. *bicolor*	KU 121341	Ecuador, Pastaza, Mera	-1.457452	-78.107976	1111
*Synophis* aff. *bicolor*	MZUTI 3529	Wild Sumaco, Ecuador	-0.675700	-77.601290	1463
*Synophis* aff. *bicolor*	MZUTI 4180	El Genairo, Ecuador	-4.166181	-78.94094	1212
*Synophis* aff. *bicolor*	UMMZ 91550	Ecuador, Napo-Pastaza, Abitagua	-1.383000	-78.083000	1482
**Western Andes**					
*Synophis* aff. *bicolor*	BMNH 1940.2.30.31	Río Solaya, Ecuador	-0.010213	-78.819510	1008
*Synophis* aff. *bicolor*	CAS 23612	Chimborazo, Naranjapata, Ecuador	-2.266667	-79.083333	763
*Synophis* aff. *bicolor*	MCZ R-164530	Ecuador, Pichincha, Tandapi	-0.419803	-78.801132	1714
*Synophis* aff. *bicolor*	QCAZ 10453	Cotopaxi: Naranjito, Bosque Integral Otonga	-0.417820	-78.988030	1655
*Synophis* aff. *bicolor*	TCWC 66209	Ecuador, Cotopaxi, Las Pampas	-0.348360	-79.076010	1238
*Synophis* aff. *bicolor*	UMMZ 185812	Ecuador, Cotopaxi, San Francisco de Las Pampas	-0.440357	-78.966629	1586
*Synophis* cf. *bicolor*	MHUA 14577	Colombia, Dpto. Antioquia, Mpio. Amalfi, V. da La Manguita, Fca. La Esperanza	6.978611	-75.044444	1394
*Synophis* cf. *bicolor*	MLS 2072	Medellin, Cordillera Central, Colombia	6.230833	-75.590556	1497
*Synophis bicolor*	MECN 6732	Tobar Donoso, Ecuador	1.189930	-78.504130	229
*Synophis bicolor*	MECN 6733	Sendero Awa, Ecuador	1.164400	-78.507120	257
*Synophis bicolor*	MZUTI 4175	Itapoa, Ecuador	0.46411	-79.15547	267
*Synophis bicolor*	UTA R-55956	Ecuador, Esmeraldas, Canton San Lorenzo	1.03212	-78.613780	318
*Synophis calamitus*	KU 164208	9 km SE Tandayapa, Pichincha Province, Ecuador	-0.047404	-78.632804	2169
*Synophis calamitus*	KU 197107	4 km SE Tandayapa, Pichincha Province, Ecuador	-0.012514	-78.650697	1889
*Synophis calamitus*	MZUTI 3694	Tambo Tanda, Ecuador	-0.020108	-78.651012	2048
*Synophis lasallei*	EPN S.974	Ecuador, Napo-Pastaza, nr. Río Talin, headwaters of the Río Bobonaza	-1.466670	-77.883300	948
*Synophis lasallei*	FHGO 6489	Ceploa, Ecuador	-1.339063	-77.670660	839
*Synophis lasallei*	FHGO 7770	Cara del Indio, Ecuador	-3.575695	-78.451020	1207
*Synophis lasallei*	FHGO 8340	El Quimi, Ecuador	-3.571852	-78.516598	752
*Synophis lasallei*	FMNH 81313	Colombia, Meta, Pico Renjifo, Serrania de la Macarena	2.476901	-73.794852	520
*Synophis lasallei*	KU 164221	2 km SSW Río Reventador, Ecuador	-0.100000	-77.600000	1479
*Synophis lasallei*	MCZ R-156873	Ecuador, Napo Prov., Inecel Station, Cascada San Rafael, Río Quijos	-0.103401	-77.585487	1290
*Synophis lasallei*	MECN 11250	Paquisha Alto, Ecuador	-3.909518	-78.487244	1660
*Synophis lasallei*	MECN 11262	El Pangui, Ecuador	-3.624502	-78.586510	814
*Synophis lasallei*	MECN 2220	Puyo, Ecuador	-1.466780	-77.983350	957
*Synophis lasallei*	MLS/CJSP	N of Alban, cen. Cundinamarca Dept., cen. Colombia	4.883333	-74.450000	1983
*Synophis lasallei*	MZUTI 4181	Sacha Yaku, Ecuador	-1.407882	-77.711092	974
*Synophis lasallei*	USNM 233061	Río Arajuno, headwaters of, tributary of Río Napo, Pastaza, Ecuador	-1.400000	-77.883300	969
*Synophis lasallei*	USNM 233062	Río Siquino, tributary of Río Villano, Upper Curaray, Pastaza, Ecuador	-1.455303	-77.714685	576
*Synophis lasallei*	USNM 233063	Río Bobonaza, headwaters of, Ecuador	-1.512156	-77.833454	594
*Synophis lasallei*	WWL 977-978	Colombia, Meta prov., Villavicencio	4.150000	-73.633333	539
*Synophis plectovertebralis*	UVC 11580	Haciendo San Pedro, 6km S El Queremal, Municipio Dagua, Valle del Cauca, Colombia	3.483333	-76.700000	1830
*Synophis zaheri*	MZUTI 3353	Buenaventura Lodge, Ecuador	-3.647970	-79.755070	874
*Synophis zaheri*	MZUTI 3355	Buenaventura Lodge, Ecuador	-3.648820	-79.756400	812

**Table 3.** Summary of measured diagnostic characters (external meristic features) for diaphorolepidine species. These data are a summary of Table 1 (omitting some subcaudal scale counts from apparently truncated tails), and can be used to identify ambiguous specimens in the field or collections, and should be updated with new material in the future.

Species	MT	IL	SL	PO	V	SC	D1	D2	D3
*Diaphorolepis laevis*	16	10	8–9	2	157	84	19	19	17
*Diaphorolepis wagneri*	23–25	10–13	8–9	1–3	181–197	131–141	19–21	19	17
*Emmochliophis fugleri*	16	8	8	2	140	97	19	19	19
*Emmochliophis miops*	13	8	8	1	145	93	19	19	19
*Synophis* aff. *bicolor*	24–27	10–11	8–9	2	152–166	96–122	19–21	17–19	17–18
*Synophis* cf. *bicolor*	23–24	10–12	8	2	184–193	127–131	19	19	17
*Synophis bicolor*	16	9–11	8	2	174–183	129–143	19	17–19	17
*Synophis calamitus*	–	9–11	7–9	1–2	163–166	110–125	21–23	19	17
*Synophis lasallei*	24	10–11	7–9	1–2	144–165	101–126	19–23	19–22	17–21
*Synophis plectovertebralis*	–	7–8	7–8	1	144–147	79–91	19	19	17
*Synophis zaheri*	–	8–9	8	2	166–169	111–112	19	19	17

First, the head scalation of *Diaphorolepis wagneri* has not been accurately characterized by most authors (see Bogert 1964). Additionally, the holotype of *Diaphorolepis laevis* was incorrectly described with respect to several major characters (Werner 1923). Finally, reviewing museum specimens, including most holotypes, reveals that the current species boundaries and diagnoses are oftentimes inaccurate with respect to the observed range of variation in the relevant characters. In particular, the holotype of *Synophis bicolor* does not match many populations typically referred to this species (Bogert 1964; Hillis 1990; Sheil and Grant 2001).

In the case of *Diaphorolepis wagneri*, the postoculars can range from 1–3 (rather than 1–2), as illustrated by Bogert (1964), but not discussed explicitly. Werner (1901) apparently considered the small, lower postocular to be a subocular. Occasionally, the middle postocular will not be in contact with the brille, and resembles a temporal, behind the two remaining postoculars. As noted previously, the nasals are never divided, but only creased (Sheil and Grant 2001), contrary to reports from some previous authors (Bogert 1964; Hillis 1990).

In the case of *Diaphorolepis laevis*, Werner (1923) diagnosed the species as having fewer ventrals and subcaudals than *Diaphorolepis wagneri*, and smooth dorsal scales. Examination of the holotype (NMW 14860) reveals that it is indeed keeled, albeit weakly, throughout most of the midbody and posterior dorsal scale rows. This includes a bicarinate vertebral scale row that was previously considered to be diagnostic only of *Diaphorolepis wagneri*. The specimen appears to have a lighter-colored nuchal collar, though this may be a preservation artifact. The type locality within Colombia is unknown.

In the case of *Synophis bicolor*, the holotype (MZUT 257) has 180 ventrals, 136 subcaudals, and 9 infralabials, whereas sampled populations from the Andes of Ecuador typically have 152–166 ventrals, 96–122 subcaudals, and 10 or 11 infralabials. The locality of the holotype is unknown. Sampled populations from the Chocó of Ecuador match the holotype more closely, with 174–183 ventrals, 129–143 subcaudals, and 9–11 infralabials. The Chocóan populations typically occur at low to middle elevations (~200–300m), whereas Andean populations occur at higher elevations (~800–1700m). Populations from the northern western Andes of Colombia have 184–193 ventrals, 127–131 subcaudals, and 10–12 infralabials.

These three populations (Chocóan, Colombian Andean, and Ecuadorean Andean; Figs 3D, 4), correspond to three deeply divergent genetic lineages within *Synophis bicolor* (Fig. 1). A full revision of this species complex is pending further molecular and morphological sampling. We refer to the Chocóan populations as *Synophis bicolor*, the Ecuadorean Andean populations as *Synophis* aff. *bicolor*, and the Colombian Andean populations as *Synophis* cf. *bicolor* (using aff. versus cf. somewhat arbitrarily) for the remainder of the paper. The *Synophis bicolor* group is also weakly paraphyletic with respect to the sampled specimen of *Synophis lasallei*, which is the sister lineage of the Ecuadorean Andean lineages. The specimen of *Synophis lasallei* (MZUTI 4181) strongly matches the other *Synophis lasallei* specimens examined (Table 1), and is thus not a mis-identified *Synophis bicolor*.

Finally, we report here on two specimens of *Synophis* aff. *calamitus* from low to middle elevations on the Pacific versant of the Andes in SW Ecuador. These are diagnosable from the species above based on numerous characters, and we here name them:

#### Synophis zaheri sp. n.

Taxon classificationAnimaliaSquamataColubridae

http://zoobank.org/AEE122E3-497B-4DBF-8A2B-79DDD231E42B

Figs 3 , 5 , 8

##### Holotype.

MZUTI 3353 (Fig. 3A), an adult male collected on 30 December 2013 at ~2200h by Alejandro Arteaga, Lucas Bustamante, Rita Hidalgo, Daniel Mideros, and Diana Troya, in the vicinity of Buenaventura Reserve (Fundación Jocotoco), near Piñas, El Oro Province, SW Ecuador, 874m above sea level (-3.65, -79.76; Fig. 5), in a narrow band of cloud forest on the Pacific versant of the Andes.

##### Paratype.

MZUTI 3355 (Fig. 3B), adult male collected a few minutes after the holotype, a few meters away.

##### Etymology.

Named after the preeminent Brazilian herpetologist Hussam El-Dine Zaher, for his innumerable contributions to South American herpetology and snake systematics.

##### Diagnosis.

*Synophis zaheri* can be differentiated from *Diaphorolepis* by an unmodified vertebral scale row with a single weak keel (versus a laterally expanded vertebral scale row, bicarinate or smooth); from *Emmochliophis* by the presence of a loreal (versus absence); from *Synophis bicolor* by having 166–169 ventrals (versus 174–183) and 111–112 subcaudals (versus 129–143); from *Synophis* aff. *bicolor* by having 8 or 9 infralabials (versus 10 or 11) and lighter brown dorsal coloration in life (versus darker black); from *Synophis* cf. *bicolor* by having 166–169 ventrals (versus 184–193), 111–112 subcaudals (versus 127–131), and 8 or 9 infralabials (versus 10–12); from *Synophis calamitus* by having two postoculars (versus one typically) and internasals in contact (versus divided typically); from *Synophis lasallei* by having 166–169 ventrals (versus 144–165), 19 dorsal scale rows at midbody (versus 21–23 typically), 8 or 9 infralabials (versus 10 or 11), and by having the anteriormost dorsal scale rows smooth (versus keeled); and from *Synophis plectrovertebralis* by absence of a nuchal collar (versus presence) and two postoculars (versus one).

##### Description.

Small-sized snakes (351–372mm SVL, 184–194mm TL) with slender bodies and head distinct from neck. Eye large (>1/3 head height), bulbous, and black in life, with pupil not easily distinguishable from iris. Pupil round in preservative (though this may be an effect of fixation). Dorsum coloration grayish-brown with iridescent sheen in life and preservation, no light-colored nuchal collar in adults, and posterior supralabials mostly pigmented (>50%). Ventral coloration primarily bright yellowish-white, extending onto margins of ventral scales and supralabials. Posterior one-third of ventral surface anterior to vent becomes increasingly mottled, and ventral surface of tail color of dorsum. Squamation pattern includes 166–169 ventral scales, 111–112 subcaudals, 19-19-17 dorsal scale rows (scale-row reduction of 2 rows past midbody), anal single, no apical pits, mid-body dorsal scales with weak single keel (first few dorsal scale-rows smooth), vertebral scale row not enlarged, nuchal scales smooth, 8 supralabials, 8 or 9 infralabials, 2 postoculars, loreal present, nasal undivided, fused prefrontals, internasals in contact, and rostral concave. Condition of the vertebrae, which are heavily modified in *Emmochliophis* and *Synophis* (Fritts and Smith 1969; Savitzky 1974; Hillis 1990) unknown, pending skeletal preparation or micro-CT scanning. Everted hemipenes are slightly bilobed, semicalyculate, and semicapitate, relatively stout and bulbous, covered in large spines or hooks, similar to that of *Diaphorolepis* and *Synophis* aff. *bicolor* and *Synophis lasallei* (Bogert 1964; Zaher 1999; Martinez 2011). Both specimens were active by night in primary evergreen foothill forest, with canopy cover between 70 and 100%. The holotype MZUTI 3353 was found on the ground, whereas the paratype MZUTI 3355 was found 50 cm above the ground in a bush. Neither were found close to water, but were active after a rainy day.

In light of this new species and the updated material we have located and examined (Tables 1, 2), we have prepared updated accounts for the tribe and the other species. Hopefully, these will serve as useful descriptive summaries for taxonomic boundaries, species delimitation, and the assignment of new specimens and populations to species-level groups. We focus primarily on the external morphological characters that will be of greatest use for identifying specimens in the field and from preserved collections. In some cases, more detailed information can be found in the original descriptions cited. The tribe name Diaphorolepidini was introduced in the PhD thesis of Jenner (1981), for which availability as a published work is ambiguous. We conservatively continue to credit the name to her, rather than treat it as unavailable and re-describe it ourselves.

#### Tribe Diaphorolepidini

Taxon classificationAnimaliaSquamataColubridae

Jenner, 1981

Diaphorolepis Jan, 1863 (type genus by original designation)
Emmochliophis Fritts & Smith, 1969
Synophis Peracca, 1896

##### Etymology.

Apparently from the Greek *diaphoros* for “differentiated” and *lepis* for “scales,” likely referring to the enlarged vertebral scale row as compared to the rest of the dorsal scales.

##### Description.

A group of relatively small-sized (<550mm SVL) dipsadine snakes restricted to the Darien of Panama and northern Andes of South America with fused prefrontals and either an expanded vertebral scale row (*Diaphorolepis*) or expanded zygapophyses and neural spines in adults (*Emmochliophis* and *Synophis*).

##### Notes.

The tribe name has also been spelled ‘Diaphorolepini’ by Sheehy (2012), but Diaphorolepidini is the correct spelling based on the suffix –*lepis*, for which the stem is –*lepid* + –*ini*. This is a greatly restricted definition of Diaphorolepidini over the original description (Jenner 1981), which included *Atractus*, *Chersodromus*, *Crisantophis*, *Elapomorphus*, *Enulius*, *Gomesophis*, *Pseudotomodon*, *Ptychophis*, and *Sordellina*.

#### Genus Diaphorolepis

Taxon classificationAnimaliaSquamataColubridae

Jan, 1863

Diaphorolepis laevis Werner, 1923
Diaphorolepis wagneri Jan, 1863 (type species by monotypy)

##### Etymology.

Apparently from the Greek *diaphoros* for “differentiated” and *lepis* for “scales,” likely referring to the enlarged vertebral scale row as compared to the rest of the dorsal scales.

##### Description.

Relatively small-sized (<550mm SVL) dipsadine snakes restricted to the Darien in Panama and northern Andes of South America, with 16–25 maxillary teeth, 10–13 infralabials, 8 or 9 supralabials, fused prefrontals, internasals in contact, loreal present, 1–3 postoculars, 157–197 ventrals, 84–141 subcaudals, dorsal scales in (19–21)-19-17 rows, and expanded vertebral scale row with weak to strong double keeling.

##### Notes.

This genus was validly described by Jan (1863), and re-described by Werner (1897). Werner (1901) later incorrectly deemed Jan’s name a *nomen nudum*, and re-described the genus and type species, designating a neotype. However, this was an error of interpretation, later realized by Werner himself (Werner 1929), and neither the re-description or neotype designation have any nomenclatural validity (see Bogert 1964). The lower subcaudal counts for some specimens likely represent truncated tails.

#### Diaphorolepis laevis

Taxon classificationAnimaliaSquamataColubridae

Werner, 1923

##### Holotype.

NMW 14860, locality given only as “Colombia.”

##### Etymology.

Apparently from the Latin *laevis* for “smooth,” referring to the anterior dorsal scales.

##### Description.

Relatively small-sized snake (350mm SVL) with 10 infralabials, 8/9 supralabials, 2 postoculars, internasals in contact, fused prefrontals, loreal present, nuchal collar apparently present, 16/18 maxillary teeth, 157 ventrals, 84 subcaudals, 19-19-17 dorsal scale rows, vertebral scale row is enlarged, with single keels on lateral dorsal scale rows and double keels on enlarged vertebral scale row weak to absent anteriorly and weak posteriorly. Uniformly light-colored venter and dark-colored dorsum in preservative. Nothing is known of the hemipenes or vertebrae.

##### Notes.

Known only from the type specimen. The original description states that the dorsal scales are smooth, but weak keels are evident throughout the posterior portion of the body. A specimen at Harvard, reportedly from Leticia, Amazonas, Colombia, bears the identification *Diaphorolepis laevis* (MCZ R-143839). Upon examination, this specimen is clearly not *Diaphorolepis* on the basis of divided prefrontals (versus united in *Diaphorolepis*), lack of an enlarged bicarinate vertebral scale row (versus presence), and presence of an ocellated dorsal color-pattern (versus uniformly colored dorsum). The overall resemblance is of *Dipsas* sp.

#### Diaphorolepis wagneri

Taxon classificationAnimaliaSquamataColubridae

Jan, 1863

##### Holotype.

ZSM 2708/0, locality given only as “Andes of Ecuador.” We revise this by subsequent restriction (*sensu* Smith 1953) to Milpé, Pichincha province, Ecuador (0.035, -78.87; 1076m), the locality of one of the specimens (MZUTI 3322) examined here.

##### Description.

Relatively small-sized snakes (276–524mm SVL) with 23–25 maxillary teeth, 10–13 infralabials, 8 or 9 supralabials, 1–3 postoculars with the lower occasionally resembling a subocular and the middle occasionally resembling a temporal, fused prefrontals, internasals in contact, loreal present, incomplete nuchal collar present in juveniles (MZUTI 3322) fading ontogenetically, 181–197 ventrals, 131–141 subcaudals, (19–21)-19-17 dorsal scale rows, strong keels present on dorsal scales, and enlarged, bicarinate vertebral scale row. Uniformly cream-colored venter and dark-brown to black dorsum. Lumbar vertebrae are constricted near the middle, zygapophyses and neural spines are not expanded. The hemipenis has been briefly described (Bogert 1964), but prior to modern classifications of the organ (Zaher 1999), and needs to be examined in more detail. Ranges at low to middle elevations (~300–1600m) along the Pacific versant from the Darien in Panama to central Ecuador.

##### Etymology.

Most likely after Moritz Wagner, who collected the holotype (see Bauer 2013), and not Johann Andreas Wagner as suggested by previous authors (Beolens et al. 2011).

##### Notes.

The re-description and neotype designation (NMW 18915) of Werner (1901) have no nomenclatural validity (see Bogert 1964).

#### Genus Emmochliophis

Taxon classificationAnimaliaSquamataColubridae

Fritts & Smith, 1969

Emmochliophis fugleri Fritts & Smith, 1969 (type species by monotypy)
Emmochliophis miops (Boulenger, 1898)

##### Etymology.

From the Greek *emmochlion* for “a socket for a bar” and *ophis* for “snake,” referring to the unique interlocking vertebrae (Fritts and Smith 1969).

##### Description.

Relatively small-sized (~250mm SVL) terrestrial snakes restricted to the Pacific Andean slopes of NW Ecuador, with a small number (<17) of maxillary teeth, 8 supralabials, 8 infralabials, fused prefrontals, internasals in contact, loreal absent, fewer than 150 ventrals, fewer than 100 subcaudals, dorsal scales in 19 rows without reduction, trunk vertebrae with lateral expansion of the zygapophyses, and expanded zygapophyses forming a rod-and-groove mechanism in *Emmochliophis fugleri*, but not in *Emmochliophis miops*.

##### Notes.

Both species are known only from the types. The hemipenis of *Emmochliophis fugleri* has been briefly described (Fritts and Smith 1969), but prior to modern classifications of the organ (Zaher 1999), and needs to be examined in more detail. The organ is unknown in *Emmochliophis miops*, as the sole known specimen is female (Sheil 1998).

#### Emmochliophis fugleri

Taxon classificationAnimaliaSquamataColubridae

Fritts & Smith, 1969

##### Holotype.

UIMNH 78795, 4 km. E Río Baba bridge, 24 km. S Santo Domingo de los Colorados, Pichincha, Ecuador, ~600 m.

##### Etymology.

After Dr. Charles Fugler, who collected the holotype.

##### Description.

A terrestrial snake from the Pacific Andean slopes of NW Ecuador, diagnosable by 16 maxillary teeth, 8 infralabials, 8 supralabials, 2 postoculars, internasals in contact, loreal absent, nuchal collar absent, 140 ventrals, 97 subcaudals, dorsal scales in 19 rows without reduction, strong keels, and zygapophyses expanded laterally forming rod–and–bar assembly. Type locality is surrounded by banana plantations. Little else is known about the habits or habitat of the species.

##### Notes.

Known only from the type specimen, a male, collected by C. Fugler in February 1966.

#### Emmochliophis miops

Taxon classificationAnimaliaSquamataColubridae

(Boulenger, 1898)

Synophis miops Boulenger, 1898

##### Holotype.

BMNH 1946.1.12.30, Paramba, Ecuador (=Parambas, Imbabura fide Lynch and Duellman 1997)

##### Etymology.

None given by Boulenger (1898); likely from the Greek *miops* for “myopia,” in reference the species’ small eyes, given as diagnostic by Boulenger.

##### Description.

Relatively small-sized (~250mm SVL) terrestrial snake from the Pacific Andean slopes of NW Ecuador, diagnosable by 13 maxillary teeth, 8 infralabials, 8 supralabials, 1 postocular, internasals in contact, loreal absent, nuchal collar present, 145 ventrals, 93 subcaudals, dorsal scales in 19 rows without reduction, strong keels, and lateral expansion of the zygapophyses. Type locality is humid subtropical lower montane forest. Little else is known about the habits or habitat of the species. Stomach of type specimen contains remains of a gymnophthalmid lizard (Sheil 1998).

##### Notes.

Known only from the type specimen, a female, collected by W. F. H. Rosenberg in October 1897. The type specimen was re-described in great detail by Sheil (1998).

#### Genus Synophis

Taxon classificationAnimaliaSquamataColubridae

Peracca, 1896

Synophis bicolor Peracca, 1896 (type species by monotypy)
Synophis calamitus Hillis, 1990
Synophis lasallei (Nicéforo-Maria, 1950)
Synophis plectovertebralis Sheil & Grant, 2001
Synophis zaheri Pyron, Guayasamin, Peñafiel, Bustamante, & Arteaga, 2015

##### Etymology.

None given by Peracca (1896); presumably from the Greek *syn*- for “with” or “together” and *ophis* for “snake,” though the intended meaning of “with snake” is unclear.

##### Description.

Relatively small-sized (~300mm SVL) dipsadine snakes of the Andes and Chocó of Colombia and Ecuador, with 16–27 maxillary teeth, 7–11 infralabials, 7–9 supralabials, fused prefrontals, loreal present, 1 or 2 postoculars, 144–184 ventrals, 88–138 subcaudals, dorsal scales in (19–21)-(17–21)-(17–20) rows, neural spine expanded and flattened, laterally expanded zygapophyses, and hemipenes slightly bilobed, semicalyculate, and semicapitate, relatively stout and bulbous, covered in large spines or hooks.

##### Notes.

On the basis of similar scale counts, but apparently without examining specimens, Amaral (1929) considered the holotype of *Synophis bicolor* (at the time, the only known specimen from the only known species) to be synonymous with *Diaphorolepis wagneri*. These snakes are extremely rare, accounting for the paucity of knowledge and unclear species-boundaries. Numerous undescribed species from many new localities are known, and await description (*pers. comm.*, T. Grant, E. Meneses-Pelayo, O. Torres-Carvajal, and J. Arredondo).

#### Synophis bicolor

Taxon classificationAnimaliaSquamataColubridae

Peracca, 1896

##### Holotype.

MZUT 257, locality given only as “South America.”

##### Etymology.

None given by Peracca (1896); presumably from the Greek *bi-color* for “two colors,” referring to the dark dorsum and light venter.

##### Description.

Small-sized (~200–400mm SVL) dipsadine snakes of the Andes and Chocó of Colombia and Ecuador, diagnosable by 16–27 maxillary teeth, 9–12 infralabials, 8 or 9 supralabials, fused prefrontals, loreal present, 2 postoculars, 152–193 ventrals, 96–143 subcaudals, dorsal scales in (19–21)-(17–19)-(17–18) weakly keeled rows, neural spine expanded and flattened, laterally expanded zygapophyses, and hemipenes slightly bilobed, semicalyculate, and semicapitate, relatively stout and bulbous, covered in large spines or hooks. Populations of this species are found in both lowland Chocóan rainforest and Andean cloud forests. Individuals are often found in leaf litter or in bushes, active at night. One collection from the Pacific Andean slopes of Ecuador (UMMZ 185886–185891) represents clutches of 2, 2, and 8 eggs, with hatchlings 125–132mm SVL. Nothing is known of diet.

##### Notes.

This is a species complex comprising at least three species-level taxa, which are distinct genetically, geographically, and morphologically (Figs 1, 3D, 4, 7, 9; Tables 1–3).

First are the Ecuadorean Andean highlands populations (*Synophis* aff. *bicolor*), which occur both on both the Pacific and Andean versants (~800–1700m). These are diagnosable by number of ventrals (152–166), subcaudals (96–122), infralabials (10 or 11), and supralabials (8 or 9), in combination. One individual (UMMZ 91550) has 24/27 maxillary teeth. The southernmost individual we examined (MZUTI 4180) has a very low number of ventral scales (152) compared to the remaining populations (160–166). Populations east and west of the Andes may also be a distinct species (O. Torres-Carvajal, *pers. comm.*), and are presented separately here. Most records from the Pacific versant north of the Río Toachi appear to represent *Synophis calamitus* (see below); one specimen reported from north of the river (BMNH 1940.2.30.31) may be mis-labeled, mis-identified, or the locality mis-referenced, or the species may be sympatric at some localities north of the river.

Second are the Chocóan populations from NW Ecuador, and presumably SW Colombia (~200–300m). These match the holotype in having 174–183 ventrals, 129–138 subcaudals, 8 supralabials, and typically 9 infralabials, though one specimen from further south (MZUTI 4175) has 11. We revise the type locality of *Synophis bicolor* by subsequent restriction (*sensu* Smith 1953) to Tobar Donoso, Carchi Province, Ecuador (1.19, -78.50), locality of several specimens examined here (Tables 1, 2; Figs 1, 4, 7, 9), to cement this association. Thus, this population represents *Synophis bicolor* *sensu stricto* in the case of future revision.

Third are the Colombian Andean highland populations (~1400–1500m; see Nicéforo-Maria 1970), which differ from the holotype in having 184–193 ventrals (versus 180), 127–131 subcaudals (versus 136), and 10–12 infralabials (versus 9). This group likely represents a third species, *Synophis* cf. *bicolor*. While we refrain from describing these additional *Synophis bicolor*-group species here based on limited current sampling, the populations described above likely represent at least two (Ecuadorean Andean highland and Colombian Andean Highland) if not three (E and W Ecuadorean and Colombian Andean highland) species.

#### Synophis calamitus

Taxon classificationAnimaliaSquamataColubridae

Hillis, 1990

##### Holotype.

KU 197107, 4 km SE Tandayapa, Pichincha Province, Ecuador.

**Paratype.** KU 164208, 9km SE Tandayapa, Pichincha Province, Ecuador.

##### Etymology.

From the Latin for “calamity,” referring to accidents that befell the original collectors (Hillis 1990).

##### Description.

A group of relatively small (~450mm SVL) dipsadine snakes of the cloud forests of the Pacific versant of the Andean highlands of Ecuador diagnosable by 9–11 infralabials, 7–9 supralabials, fused prefrontals, internasals separated, loreal present, 1 or 2 postoculars, 163–166 ventrals, 110–125 subcaudals, dorsal scales in (21–23)-19-17 weakly keeled rows, neural spine expanded and flattened, and laterally expanded zygapophyses. Known from middle to high-elevation (~1900–2200m) cloud forests north of the Río Toachi. Nothing is known of diet or reproduction.

##### Notes.

A detailed description was also provided by Hillis (1990). The hemipenes have likely not been examined. Easily confused with *Synophis bicolor*; at least one specimen (QCAZ 11931) from near the type locality was originally mis-identified (O. Torres-Carvajal, *pers. comm.*). We suggest that all populations north of the Río Toachi are likely to represent *Synophis calamitus*. As mentioned above, one specimen apparently matching *Synophis bicolor* (BMNH 1940.2.30.31) is known from Río Soloya near Mindo north of Río Toachi, but this may have been mis-labeled, or mis-referenced geographically. The specimen of *“Synophis bicolor”* examined by Zaher (1999), QCAZ 452, cannot be located (O. Torres-Carvajal, *pers. comm.*), but originates from Chiriboga, Pichincha Province, Ecuador, north of Río Toachi, and thus may represent an *Synophis calamitus*. If this is the case, the hemipenes of *Synophis calamitus* and *Synophis lasallei* are nearly identical (Zaher 1999; Martinez 2011). Finally, one specimen sequenced here from Tambo Tanda (MZUTI 3694) appears to have aberrantly subdivided head scales, possessing one extra postocular, and 2 extra supralabials and infralabials (Fig. 8), which are misshapen and abnormally small. The badly damaged paratype also appears to have two postoculars on one side (O. Torres-Carvajal, *pers. comm.*). Thus, we concur with Hillis (1990) that one postocular, 7 or 8 supralabials, and 9 infralabials (along with the divided internasals and smooth anterior dorsal scale-rows) are generally diagnostic of the species, but with rare individual variation.

#### Synophis lasallei

Taxon classificationAnimaliaSquamataColubridae

(Nicéforo-Maria, 1950)

Diaphorolepis lasallei Nicéforo-Maria, 1950

##### Holotype.

MLS/CJSP uncat., from N of Albán, cen. Cundinamarca Dept., cen. Colombia.

##### Etymology.

After the Instituto de La Salle, in Bogotá (Nicéforo-Maria 1950).

##### Description.

Smaller (~300mm SVL) dipsadine snakes of the Amazonian versant of the Andes of Ecuador and Colombia, diagnosable by 24 maxillary teeth, 10 or 11 infralabials, 7–9 supralabials, fused prefrontals, internasals in contact, loreal present, 1 or 2 postoculars, nuchal collar absent, 144–165 ventrals, 101–126 subcaudals, dorsal scales in (19–23)-(19–22)-(17–21) strongly keeled rows even on head and neck, venter dark in some populations, neural spines expanded and flattened, and laterally expanded zygapophyses. Known from low to high elevations (~500–2000m) along the Amazonian versant of the Andes from central Colombia to central Ecuador. Nothing is known of diet or reproduction.

##### Notes.

The hemipenes are very similar to both *Diaphorolepis* and *Synophis bicolor* (Bogert 1964; Zaher 1999; Martinez 2011). Much like *Synophis bicolor*, this species as currently described has a large geographic and elevational range, with wide variation in phenotype. There is significant variation in the number of dorsal scale rows and reduction thereof. One specimen from Ecuador (MCZ R-156873) has only one postocular and 7 supralabials, but otherwise matches the species. All other specimens have 2 and 8, respectively. Another specimen from Ecuador (MECN 2220) has 165 ventrals and 117 subcaudals with 19-19-17 scale rows, and is thus indistinguishable from *Synophis* aff. *bicolor*, with the exception of the strong keels on the nuchal scales and geographic distance from the nearest highland populations of *Synophis* aff. *bicolor*. All other specimens of *Synophis lasallei* have 144–156 ventrals, and most have (21–23)-(21–22)-(19–21) dorsal scale rows. Thus, it seems exceptionally likely that this is a species complex, possibly divided between highland and lowland, or northern and southern populations.

#### Synophis plectovertebralis

Taxon classificationAnimaliaSquamataColubridae

Sheil & Grant, 2001

##### Holotype.

UVC 11858, from Hacienda San Pedro, about 6 km south El Queremal, Municipio Dagua, Departamento del Valle del Cauca, Colombia.

##### Paratype.

UVC 11580, from type locality.

##### Etymology.

From the Latin *plecto*- for “braided” or “woven” and *veretbralis* for “vertebrae,” referring to the appearance of the interlocking zygapophyses viewed from above (Sheil and Grant 2001).

##### Description.

Relatively small (~200mm SVL) dipsadine snakes of the Pacific versant of the Andean Highlands of W Colombia, diagnosable by 24 maxillary teeth, 7 or 8 infralabials, 7 or 8 supralabials, fused prefrontals, internasals in contact, loreal present, 1 postocular, nuchal collar present, 144–147 ventrals, 79–91 subcaudals, dorsal scales in 19-19-17 weakly keeled rows, neural spines expanded and flattened, and laterally expanded zygapophyses forming a partially interlocking complex. The type locality is a middle elevation (~1800m) cloud forest. Both known specimens were collected in moist leaf litter; one was active at night. The stomach of the holotype contained a *Ptychoglossus stenolepis* (Sauria: Gymnophthalmidae).

##### Notes.

Known only from the holotype and paratype (apparently juveniles), though other material has apparently been collected in Colombia, near the type locality (T. Grant and E. Meneses-Pelayo, *pers. comm.*). The hemipenes have not been examined. A more detailed description of the two specimens is provided by Sheil and Grant (2001).

Given our restriction of the name, we also provide the following re-description of the re-delimited Nothopsini. Note that we have not performed a comparative examination of a large series of preserved material, and these data are summarized from the literature (Dunn and Dowling 1957; Savage 2002; Kohler 2008; McCranie 2011) to provide a basis for future revisions.

#### Tribe Nothopsini

Taxon classificationAnimaliaSquamataColubridae

Cope, 1871

Nothopsis Cope, 1871 (type genus by monotypy)
Nothopsis rugosus Cope, 1871
Nothopsis affinis Boulenger, 1895 (Holotype BMNH 1946.1.15.62, “Salidero, NW Ecuador, 350ft”) [subjective junior synonym of *Nothopsis rugosus* fide Dunn & Dowling 1957]
Nothopsis torresi Taylor, 1951 (Holotype KU 28710, “’Morehead’ Finca, 5 miles southwest of Turrialba, Costa Rica”) [subjective junior synonym of *Nothopsis rugosus* fide Dunn & Dowling 1957]

##### Holotype.

USNM 12427, type locality “Isthmus of Darien [Panama]”

##### Etymology.

From the Greek *nothos* for “bastard” and *opsis* for “appearance,” with Cope (1871) apparently referring to putative mimicry of *Bothrops atrox*.

##### Description.

A relatively small-sized (<350mm SVL) dipsadine snake, ranging in Central and South America from Honduras to Colombia and Ecuador, in lowland and middle-elevation rainforests, 250-900m, distinguishable from nearly all other similar or related snakes in the area by the rugose, granular nature of the dorsal scales, in particular lacking differentiation of the cephalic scales with the exception of well-defined internasals and poorly defined frontal and parietals, which are separated by rows of irregular, undifferentiated scales. Color pattern consists of irregular and poorly defined blotches of blackish or light, dark, and yellowish brown. With respect to the characters described here for diaphorolepidine species, *Nothopsis rugosus* typically exhibits 19–21 maxillary teeth, 9–13 supralabials, 11–16 infralabials, 149–162 ventrals, 81–112 subcaudals, dorsal scales in (24–30)-(26–30)-(22–26) rows, SVL of 151–320mm, and tail length of 61–133mm (see Dunn and Dowling 1957).

##### Notes.

This taxon has historically been divided up into as many as three species (see Dunn and Dowling 1957), though only a single species is currently recognized. There may be cryptic variation or undiscovered diversity within this group. Note that the family name was originally spelled Nothopidae by Cope (1871), but –*ops*– is the correct stem from –*opsis*, and Nothopsidae (and Nothopsini) is thus the correct spelling, as adopted by later authors.

## Discussion

### Systematics of Diaphorolepidini and Nothopsini

Corroborating previous results, we find that current supra-generic classification in Dipsadinae does not accurately reflect the phylogeny and describe natural groups in many cases (Pyron et al. 2011; Grazziotin et al. 2012). Support for monophyly and placement of many genera is low, and many other genera are apparently non-monophyletic. Efforts to clarify this situation are underway, sampling more taxa and characters (F. Grazziotin, *pers. comm.*). Only ~250 out of ~900 dipsadine species (Wallach et al. 2014) are sampled here for a few genes, but cryptic and undiscovered diversity is likely much higher in the group, and will require extensive additional sampling of taxa and characters to arrive at a stable phylogenetic and taxonomic resolution. The taxonomy of Dipsadinae has been contentious for quite some time (Cadle 1984a,b,c; Zaher 1999; Zaher et al. 2009; Grazziotin et al. 2012; Sheehy 2012), and will likely require extensive additional sampling of taxa and characters to provide a stable taxonomic resolution.

In particular, we find that Nothopsini is not monophyletic as historically defined, but that *Nothopsis* is strongly nested within a primarily Central American clade, with *Imantodes* and *Leptodeira*. We restrict tribe Nothopsini Cope, 1871 to *Nothopsis*. We resurrect and re-delimit Diaphorolepidini Jenner, 1981 to include only *Diaphorolepis*, *Emmochliophis*, and *Synophis*. Whereas *Emmochliophis* remains unsampled in the molecular phylogeny, it appears to be the sister-taxon of *Synophis* based on morphological data (Hillis 1990). However, our phylogeny suggests that many of the morphological characters previously used to define supra-generic groups in Dipsadinae (see Savitzky 1974; Wallach 1995) are subject to strong and rapid convergence. Thus, future studies may find an alternative placement for this genus. Finally, the genus *Xenopholis* is weakly nested within a primarily South American clade, and remains Dipsadinae *incertae sedis*.

### Species limits in Diaphorolepidini

Larger sample sizes reveal expanded ranges of diagnostic characters previously used to delimit species in Diaphorolepidini. These will hopefully assist future researchers in describing new taxa, and re-delimiting species boundaries. In particular, both *Synophis bicolor* and *Synophis lasallaei* may comprise multiple distinct species. Additional DNA sequencing and meristic and mensural measurements of more specimens should help clarify taxonomic boundaries.

In the case of *Synophis bicolor*, the Chocóan populations in Ecuador and presumably nearby Colombia match the description of the holotype, and thus likely represent the source of the original specimen, which remains to be re-described in detail. Contrastingly, highland populations in the Andean Highlands of Ecuador and Colombia are morphologically and genetically distinct, and both likely represent undescribed species. In the Ecuadorean Andes, populations of this taxon occur on both the Pacific and Amazonian versants, which may also be distinct from each other. The sampled specimen of *Synophis lasallei* is weakly nested within the sampled specimens of *Synophis bicolor*. A wide range of squamation and color pattern is observed in *Synophis lasallei*, which may represent cryptic species, as well as potential mis-identification of examined specimens. Finally, a cloud-forest population from the Pacific versant in SW Ecuador represents a new species described here as *Synophis zaheri*, allied to *Synophis calamitus*. Understanding the geographic distribution and genetic diversity in these taxa will require additional genetic sampling, which is hampered by the rarity of these species.

One of the most distinctive features of diaphorolepidine species is the highly modified condition of the vertebrae, in which the prezygapophyses and postzygapophyses are broadly expanded, forming ridges, and occasionally interlocking (Bogert 1964; Fritts and Smith 1969; Hillis 1990). Given the difficulty of preparing the skeletal material and the extreme rarity of specimens, this was not examined for *Synophis zaheri* or any additional specimens examined here. However, this may be a crucial character for future systematic revisions in the group, possibly utilizing micro-CT scanning or radiography.

Another possible source of information for delimiting species are the hemipenes. The organs are highly similar in *Diaphorolepis* and most *Synophis* species (Bogert 1964; Jenner 1981; Hillis 1990; Zaher 1999). Our observations agree with previous authors that the hemipenes are not strongly differentiated among species, though larger comparative series may reveal characters that serve to better diagnose species-level groups. In particular, the hemipenes are “nearly identical” in *Synophis bicolor* and *Synophis lasallei* (Zaher 1999; Martinez 2011), and our examination of *Synophis zaheri* shows no obvious qualitative differences. It is possible that speciation is primarily ecological or allopatric in this group, and thus there is little physical reproductive isolation.

## Conclusions

Higher-level taxonomy in Dipsadinae is still partially unresolved, and many genera and supra-generic groups are either non-monophyletic, or poorly supported and weakly placed. This includes Nothopsini Cope, 1871, which must be restricted to *Nothopsis*, if it is used at all. We resurrect and re-delimit Diaphorolepidini Jenner, 1981 to include only *Diaphorolepis*, *Emmochliophis*, and *Synophis*. The genus *Xenopholis* remains Dipsadinae *incertae sedis*. Revised and expanded diagnoses in Diaphorolepidini support the distinctiveness of all currently recognized taxa. Cryptic species are likely present in *Synophis bicolor* and *Synophis lasallei.* A new population from the cloud forest of SW Ecuador is morphologically and genetically distinct, and we here name it *Synophis zaheri*. We hope that these data will provide a robust platform for future researchers to examine species boundaries in Diaphorolepidini, as additional work clearly remains to be done. This is hampered, however, by the extreme rarity of these species.

## Supplementary Material

XML Treatment for Synophis zaheri

XML Treatment for Diaphorolepidini

XML Treatment for Diaphorolepis

XML Treatment for Diaphorolepis laevis

XML Treatment for Diaphorolepis wagneri

XML Treatment for Emmochliophis

XML Treatment for Emmochliophis fugleri

XML Treatment for Emmochliophis miops

XML Treatment for Synophis

XML Treatment for Synophis bicolor

XML Treatment for Synophis calamitus

XML Treatment for Synophis lasallei

XML Treatment for Synophis plectovertebralis

XML Treatment for Nothopsini

## Appendix I

**Table:** GenBank accession numbers for Dipsadinae and outgroup species analyzed here.

Species	12S	16S	CYTB	ND4	CMOS
*Adelphicos quadrivirgatum*	-	-	GQ895853	-	GQ895796
*Alsophis antiguae*	AF158455	AF158524	-	-	-
*Alsophis antillensis*	FJ416691	FJ416702	FJ416726	FJ416800	-
*Alsophis manselli*	-	AF158528	FJ416727	FJ416801	-
*Alsophis rijgersmaei*	FJ416697	FJ416708	FJ416729	FJ416803	-
*Alsophis rufiventris*	FJ416698	FJ416709	FJ416730	FJ416804	-
*Alsophis sajdaki*	-	-	FJ416731	FJ416805	-
*Alsophis sibonius*	FJ416692	FJ416703	FJ416728	FJ416802	-
*Amastridium sapperi*	-	-	GQ334479	GQ334580	-
*Apostolepis albicollaris*	JQ598793	JQ598856	-	-	JQ598965
*Apostolepis assimilis*	GQ457781	GQ457724	-	-	GQ457843
*Apostolepis cearensis*	JQ598794	JQ598857	-	-	JQ598966
*Apostolepis dimidiata*	GQ457782	GQ457725	JQ598917	-	GQ457844
*Apostolepis flavotorquata*	JQ598795	JQ598858	GQ895854	-	GQ895798
*Arrhyton dolichura*	AF158438	AF158507	FJ416721	FJ416795	-
*Arrhyton procerum*	AF158452	AF158521	FJ416723	FJ416797	-
*Arrhyton redimitum*	AF158439	AF158508	FJ416720	FJ416794	-
*Arrhyton supernum*	AF158436	AF158505	FJ416718	FJ416792	-
*Arrhyton taeniatum*	AF158453	AF158522	FJ416717	FJ416791	-
*Arrhyton tanyplectum*	AF158446	AF158516	FJ416722	FJ416796	-
*Arrhyton vittatum*	AF158437	AF158506	FJ416719	FJ416793	-
*Atractus* aff. *iridescens* MZUTI4122	-	KT944037	KT944049	KT944056	-
*Atractus albuquerquei*	GQ457783	GQ457726	JQ598918	-	GQ457845
*Atractus badius*	AF158425	AF158485	-	-	-
*Atractus duboisi* MZUTI62	-	KT944041	-	KT944059	-
*Atractus dunni* MZUTI2650	-	KT944038	KT944050	KT944057	-
*Atractus elaps*	-	-	EF078536	EF078584	-
*Atractus flammigerus*	AF158402	AF158471	-	-	-
*Atractus gigas* MZUTI3286	-	KT944043	KT944053	KT944061	-
*Atractus iridescens* MZUTI3758	-	-	KT944052	-	-
*Atractus iridescens* MZUTI3759	-	KT944039	KT944051	KT944058	-
*Atractus major* ANF1545	-	KT944045	-	-	-
*Atractus resplendens* MZUTI3996	KT944036	KT944042	KT944055	KT944060	-
*Atractus reticulatus*	JQ598798	JQ598886	-	-	JQ598970
*Atractus schach*	JQ598799	AF158486	-	-	JQ598971
*Atractus* sp. MZUTI4178	-	KT944040	-	-	KT944066
*Atractus trihedrurus*	GQ457784	GQ457727	JQ598919	-	GQ457846
*Atractus typhon* MZUTI3284	-	KT944044	KT944054	KT944062	-
*Atractus wagleri*	-	-	GQ334480	GQ334581	-
*Atractus zebrinus*	JQ598800	JQ598861	-	-	JQ598972
*Atractus zidoki*	AF158426	AF158487	-	-	-
*Boiruna maculata*	GQ457785	JQ598862	GQ895855	-	GQ895799
*Borikenophis portoricensis*	FJ416696	AF158517	AF471085	U49308	AF471126
*Borikenophis variegatus*	FJ416700	FJ416711	FJ416734	FJ416808	-
*Caaeteboia amarali*	GQ457807	GQ457747	JQ598921	-	GQ457867
*Calamodontophis paucidens*	GQ457786	GQ457728	-	-	GQ457848
*Caraiba andreae*	AF158442	AF158511	FJ416743	FJ416817	-
*Carphophis amoenus*	AY577013	AY577022	AF471067	-	DQ112082
*Carphophis vermis*	-	-	KP765656	-	-
*Clelia clelia*	AF158403	AF158472	-	-	JQ598973
*Coluber constrictor*	L01765	L01770	EU180432	AY487040	AY486937
*Coniophanes fissidens*	-	-	EF078538	EF078586	-
*Conophis lineatus*	GQ457788	JQ598865	JQ598924	-	JQ598975
*Conophis vittatus*	-	-	GQ895861	-	GQ895805
*Contia longicaudae*	-	-	GU112407	GU112427	-
*Contia tenuis*	AY577021	AY577030	GU112401	AF402658	AF471134
*Crisantophis nevermanni*	GU018152	GU018169	-	-	-
*Cryophis hallbergi*	-	-	GQ895863	EF078544	GQ895807
*Cubophis cantherigerus*	AF158405	AF158475	AF544669	FJ416818	AF544694
*Cubophis caymanus*	FJ416693	FJ416704	FJ416745	FJ416820	-
*Cubophis fuscicauda*	FJ416695	FJ416706	FJ416747	FJ416822	-
*Cubophis ruttyi*	FJ416699	FJ416710	FJ416746	FJ416821	-
*Cubophis vudii*	AF158443	AF158512	FJ416744	FJ416819	-
*Diadophis punctatus*	AF544765	AY577024	EU193700	EU193987	AF471122
*Diaphorolepis wagneri* MZUTI3322	-	KR814752	-	KR814775	KR814764
*Diaphorolepis wagneri* MZUTI3752	-	KR814753	-	KR814777	KR814766
*Diaphorolepis wagneri* MZUTI3901	-	KR814754	-	KR814778	KR814767
*Dipsas albifrons*	JQ598803	JQ598866	JQ598925	-	-
*Dipsas articulata*	JQ598804	JQ598867	-	-	-
*Dipsas catesbyi*	JQ598805	Z46496	JQ598926	EF078585	JQ598977
*Dipsas indica*	GQ457789	GQ457730	-	-	GQ457850
*Dipsas neivai*	GQ457790	GQ457731	-	-	GQ457851
*Dipsas pratti*	-	-	GQ334482	GQ334583	-
*Dipsas variegata*	AF158406	AF158476	-	-	-
*Drepanoides anomalus*	GQ457791	GQ457732	GQ895866	-	GQ895810
*Echinanthera melanostigma*	JQ598806	GU018174	JQ598928	-	-
*Echinanthera undulata*	JQ598807	JQ598870	JQ598929	-	JQ598978
*Elapomorphus quinquelineatus*	GQ457794	GQ457735	JQ598930	-	GQ457855
*Erythrolamprus aesculapii*	GQ457795	GQ457736	GQ895871	-	GQ895814
*Erythrolamprus almadensis*	JQ598808	JQ598871	-	-	JQ598979
*Erythrolamprus atraventer*	JQ598809	JQ598872	-	-	JQ598980
*Erythrolamprus breviceps*	AF158464	AF158533	-	-	-
*Erythrolamprus ceii*	JQ598810	JQ598873	-	-	JQ598981
*Erythrolamprus cursor*	JX905310	JX905314	-	-	-
*Erythrolamprus epinephelus*	GU018158	GU018176	-	-	-
*Erythrolamprus jaegeri*	GQ457809	GQ457749	-	-	GQ457869
*Erythrolamprus juliae*	AF158445	AF158514	-	-	-
*Erythrolamprus miliaris*	JQ598811	AF158480	JQ598931	-	JQ598982
*Erythrolamprus mimus*	GU018157	GU018175	-	-	-
*Erythrolamprus poecilogyrus*	JQ598812	JQ598875	-	-	-
*Erythrolamprus pygmaeus*	GU018154	GU018172	-	-	-
*Erythrolamprus reginae*	JQ598813	JQ598876	-	-	JQ598983
*Erythrolamprus typhlus*	GQ457811	GQ457751	-	-	GQ457871
*Farancia abacura*	Z46467	Z46491	U69832	DQ902307	AF471141
*Farancia erytrogramma*	AY577017	AY577026	KP765663	-	-
*Geophis carinosus*	-	-	GQ895872	-	GQ895815
*Geophis dubius*	-	-	KC917319	-	-
*Geophis godmani*	JQ598814	JQ598877	JQ598932	-	-
*Geophis juarezi*	-	-	KC917315	-	-
*Geophis latifrontalis*	-	-	KC917322	-	-
*Geophis occabus*	-	-	KC917323	-	-
*Geophis turbidus*	-	-	KC917321	-	-
*Gomesophis brasiliensis*	GQ457796	GQ457737	-	-	-
*Haitiophis anomalus*	FJ666091	FJ666092	-	-	-
*Helicops angulatus*	GQ457797	GQ457738	AF471037	-	AF471160
*Helicops carinicaudus*	JQ598815	-	-	-	JQ598984
*Helicops gomesi*	GQ457798	GQ457739	-	-	GQ457858
*Helicops hagmanni*	JQ598816	JQ598878	-	-	JQ598985
*Helicops infrataeniatus*	GQ457799	GQ457740	JQ598933	-	GQ457859
*Heterodon nasicus*	GQ457801	AY577027	KP765664	-	GQ457861
*Heterodon platirhinos*	AY577019	AY577028	GU112412	AF402659	JQ598986
*Heterodon simus*	AY577020	AY577029	AF217840	DQ902310	AF471142
*Hydrodynastes bicinctus*	GQ457802	GQ457742	JQ598935	-	GQ457862
*Hydrodynastes gigas*	GQ457803	GQ457743	GQ895873	-	GQ895816
*Hydromorphus concolor*	-	-	GQ895874	-	GQ895817
*Hydrops triangularis*	GQ457804	GQ457744	AF471039	-	AF471158
*Hypsiglena affinis*	-	-	GU353241	EU363055	-
*Hypsiglena chlorophaea*	EU728577	EU728577	EU728577	EU728577	-
*Hypsiglena jani*	EU728592	EU728592	EU728592	EU728592	-
*Hypsiglena ochrorhyncha*	EU728578	EU728578	EU728578	EU728578	-
*Hypsiglena slevini*	EU728584	EU728584	EU728584	EU728584	-
*Hypsiglena tanzeri*	-	-	EU728588	EU363044	-
*Hypsiglena torquata*	EU728591	EU728591	EU728591	EU728591	AF471159
*Hypsirhynchus callilaemus*	AF158440	AF158509	FJ416737	FJ416811	-
*Hypsirhynchus ferox*	AF158447	AF158515	GQ895875	FJ416816	GQ895818
*Hypsirhynchus funereus*	AF158451	AF158520	FJ416739	FJ416813	-
*Hypsirhynchus parvifrons*	AF158441	AF158510	FJ416740	FJ416814	-
*Hypsirhynchus polylepis*	AF158450	AF158519	FJ416738	FJ416812	-
*Hypsirhynchus scalaris*	AF158449	AF158518	FJ416741	FJ416815	-
*Ialtris dorsalis*	AF158456	AF158525	FJ416735	FJ416809	-
*Ialtris haetianus*	AF158458	AF158527	FJ416736	FJ416810	-
*Imantodes cenchoa*	EU728586	EU728586	EU728586	EU728586	GQ457865
*Imantodes chocoensis*	-	-	KC176250	-	-
*Imantodes gemmistratus*	-	-	GQ334487	EF078557	-
*Imantodes inornatus*	-	-	GQ334489	EF078559	-
*Imantodes lentiferus*	AF158463	AF158532	KC176252	EF078561	-
*Leptodeira annulata*	GQ457806	GQ457746	FJ416713	FJ416787	AF544690
*Leptodeira bakeri*	-	-	GQ334518	GQ334618	-
*Leptodeira frenata*	-	-	EF078532	EF078580	-
*Leptodeira maculata*	-	-	GQ334524	GQ334623	-
*Leptodeira nigrofasciata*	-	-	GQ334526	EF078581	-
*Leptodeira polysticta*	EU728590	EU728590	EU728590	EU728590	-
*Leptodeira punctata*	-	-	EF078530	EF078577	-
*Leptodeira rubricata*	-	-	GQ334527	GQ334631	-
*Leptodeira septentrionalis*	GU018148	GU018163	KC176243	KC176255	-
*Leptodeira splendida*	-	-	EF078521	EF078569	-
*Leptodeira uribei*	-	-	EF078531	EF078579	-
*Lygophis anomalus*	JQ598817	JQ598879	-	-	-
*Lygophis elegantissimus*	GQ457808	GQ457748	-	-	GQ457868
*Lygophis flavifrenatus*	JQ598818	JQ598880	-	-	-
*Lygophis lineatus*	-	-	-	-	DQ469789
*Lygophis meridionalis*	GQ457810	GQ457750	-	-	GQ457870
*Lygophis paucidens*	JQ598819	-	-	-	JQ598987
*Magliophis exiguum*	FJ416694	AF158526	AF471071	FJ416798	AF471117
*Magliophis stahli*	-	-	FJ416725	FJ416799	-
*Manolepis putnami*	JQ598820	JQ598881	JQ598936	-	JQ598988
*Mussurana bicolor*	GQ457787	GQ457729	-	-	GQ457849
*Ninia atrata*	GQ457814	JQ598882	JQ598937	GQ334659	GQ457874
*Nothopsis rugosus* ASL493	GU018159	GU018177	-	-	-
*Nothopsis rugosus* MZUTI3682	-	KR814760	KR814770	KR814779	KR814768
*Oxyrhopus clathratus*	GQ457815	GQ457754	-	-	GQ457875
*Oxyrhopus formosus*	JQ598821	AF158482	-	-	-
*Oxyrhopus guibei*	JQ598822	JQ627291	JQ598938	-	JQ598989
*Oxyrhopus melanogenys*	JQ598823	AF158489	-	-	JQ598990
*Oxyrhopus petolarius*	GU018144	GU018170	GQ334554	GQ334660	-
*Oxyrhopus rhombifer*	GQ457816	GQ457755	-	-	GQ457876
*Oxyrhopus trigeminus*	JQ598824	JQ598884	JQ598939	-	-
*Paraphimophis rusticus*	JQ598802	JQ598864	JQ598923	-	JQ598974
*Phalotris bilineatus*	JQ598827	JQ598887	JQ598943	-	-
*Phalotris lativittatus*	JQ598825	JQ598885	-	-	JQ598991
*Phalotris lemniscatus*	GQ457817	GQ457756	JQ598941	-	GQ457877
*Phalotris mertensi*	JQ598826	-	-	-	-
*Phalotris nasutus*	GQ457818	GQ457757	GQ895880	-	GQ895822
*Philodryas aestiva*	GQ457819	GQ457758	-	-	GQ457879
*Philodryas agassizii*	GQ457823	GQ457762	GQ895883	-	GQ457883
*Philodryas argentea*	GQ457842	GQ457780	JQ598944	-	GQ457899
*Philodryas baroni*	JQ598828	JQ598888	-	-	-
*Philodryas georgeboulengeri*	-	-	GQ895898	-	GQ895838
*Philodryas mattogrossensis*	GQ457820	GQ457759	-	-	GQ457880
*Philodryas nattereri*	JQ598829	JQ598889	AF236806	-	JQ598992
*Philodryas olfersii*	JQ598830	AF158484	JQ598945	-	JQ598993
*Philodryas patagoniensis*	GQ457821	JQ627296	AF236808	-	GQ457881
*Philodryas psammophidea*	GU018149	GU018168	-	-	-
*Philodryas viridissima*	AF158419	AF158474	AF236807	-	-
*Phimophis guerini*	GQ457822	GQ457761	-	-	GQ457882
*Pseudalsophis dorsalis*	JQ598832	JQ598892	JQ598946	-	JQ598994
*Pseudalsophis elegans*	AF158401	AF158470	JQ598947	-	JQ598995
*Pseudoboa coronata*	GQ457824	GQ457763	-	-	GQ457884
*Pseudoboa neuwiedii*	AF158423	AF158490	GQ895884	-	GQ895825
*Pseudoboa nigra*	AF544775	GQ457764	JQ598948	-	AF544729
*Pseudoeryx plicatilis*	GQ457826	GQ457765	GQ895885	-	GQ895826
*Pseudoleptodeira latifasciata*	EU728579	EU728579	EU728579	EU728579	-
*Pseudotomodon trigonatus*	GQ457827	GQ457766	-	-	GQ457887
*Psomophis genimaculatus*	GQ457828	GQ457767	-	-	GQ457888
*Psomophis joberti*	GQ457829	GQ457768	GQ895887	-	GQ895828
*Psomophis obtusus*	JQ598836	JQ598896	-	-	-
*Ptychophis flavovirgatus*	GQ457830	GQ457769	-	-	GQ457890
*Rhachidelus brazili*	JQ598837	JQ598897	JQ598952	-	-
*Rhadinaea flavilata*	-	-	AF471078	-	AF471152
*Rhadinaea fulvivittis*	-	-	EF078539	EF078587	-
*Rodriguesophis iglesiasi*	JQ598831	JQ598891	GQ895881	-	GQ895823
*Sibon nebulatus*	EU728583	EU728583	EU728583	EU728583	AF544736
*Sibon noalamina*	-	KP209376	-	-	-
*Sibynomorphus mikanii*	GQ457832	JQ627297	JQ598954	-	GQ457892
*Sibynomorphus neuwiedi*	JQ598838	JQ598898	-	-	-
*Sibynomorphus turgidus*	JQ598839	JQ598899	-	-	-
*Sibynomorphus ventrimaculatus*	JQ598840	JQ598900	-	-	JQ598997
*Siphlophis cervinus*	JQ598841	JQ598901	GQ895888	-	JQ598998
*Siphlophis compressus*	GQ457833	GQ457772	-	-	GQ457893
*Siphlophis longicaudatus*	JQ598842	JQ598902	-	-	JQ598999
*Siphlophis pulcher*	GQ457834	GQ457773	JQ598955	-	GQ457894
*Sordellina punctata*	JQ598843	JQ598903	JQ598956	-	JQ599000
*Stichophanes ningshaanensis*	KJ719252	KJ719252	KJ719252	KJ719252	KJ638718
*Synophis bicolor* MZUTI4180	-	KT944048	-	KT944065	KT944069
*Synophis bicolor* MHUA14577	KR814751	KR814758	KR814773	-	KR814769
*Synophis bicolor* MZUTI3529	-	KR814759	KR814771	KR814780	KR814762
*Synophis bicolor* MZUTI4175	-	KT944046	-	KT944063	KT944067
*Synophis bicolor* UTA R-55956	-	-	JX398697	JX398557	-
*Synophis calamitus* KU197107	KR814622	KR814640	KR814697	KR814711	KR814663
*Synophis calamitus* MZUTI3694	-	KR814755	KR814772	KR814774	KR814765
*Synophis lasallei* MZUTI4181	-	KT944047	-	KT944064	KT944068
*Synophis zaheri* MZUTI3353	-	KR814756	-	KR814776	KR814761
*Synophis zaheri* MZUTI3355	-	KR814757	-	KR814781	KR814763
*Tachymenis peruviana*	GQ457835	GQ457774	-	-	GQ457895
*Taeniophallus affinis*	JQ598844	JQ598905	JQ598957	-	GQ457853
*Taeniophallus brevirostris*	GQ457793	GQ457734	JQ598958	-	GQ457854
*Taeniophallus nicagus*	JQ598845	JQ598906	-	-	JQ599001
*Tantalophis discolor*	-	-	EF078541	EF078589	-
*Thalesius viridis*	AF158468	AF158538	-	-	-
*Thamnodynastes hypoconia*	JQ598846	-	-	-	-
*Thamnodynastes lanei*	GQ457836	GQ457775	-	-	-
*Thamnodynastes pallidus*	GU018155	GU018166	-	-	-
*Thamnodynastes rutilus*	GQ457837	GQ457776	-	-	GQ457896
*Thamnodynastes strigatus*	JQ598847	JQ598907	JQ598959	-	-
*Thermophis baileyi*	-	-	EU864148	KF595097	EU496922
*Thermophis zhaoermii*	GQ166168	GQ166168	GQ166168	GQ166168	KF514882
*Tomodon dorsatum*	GQ457838	GQ457777	GQ895892	-	GQ895833
*Tretanorhinus nigroluteus*	-	-	GQ895893	-	GQ895834
*Trimetopon gracile*	GU018160	GU018178	-	-	-
*Tropidodipsas sartorii*	-	-	EF078540	EF078588	-
*Tropidodryas serra*	JQ598848	JQ598908	JQ598961	-	-
*Tropidodryas striaticeps*	GQ457839	GQ457778	AF236811	-	-
*Uromacer catesbyi*	AF158454	AF158523	FJ416714	FJ416788	-
*Uromacer frenatus*	AF158444	AF158513	FJ416715	FJ416789	-
*Uromacer oxyrhynchus*	FJ416701	FJ416712	FJ416716	FJ416790	-
*Xenodon dorbignyi*	GQ457812	GQ457752	-	-	GQ457872
*Xenodon guentheri*	JQ598849	JQ598909	-	-	-
*Xenodon histricus*	GQ457813	GQ457753	JQ598962	-	GQ457873
*Xenodon matogrossensis*	JQ598850	JQ598910	-	-	-
*Xenodon merremi*	GQ457840	JQ598911	JQ598963	-	GQ457898
*Xenodon nattereri*	JQ598851	JQ598912	-	-	-
*Xenodon neuwiedii*	GQ457841	GQ457779	AF236814	-	-
*Xenodon pulcher*	JQ598852	JQ598913	-	-	-
*Xenodon semicinctus*	GU018156	GU018173	GQ895877	-	-
*Xenodon severus*	JQ598853	Z46474	JQ598964	-	-
*Xenopholis scalaris* GFM825	-	JQ598915	-	-	-
*Xenopholis scalaris* JPV	GU018145	GU018164	-	-	-
*Xenopholis scalaris* KU222204	-	-	GQ895897	-	GQ895837
*Xenopholis scalaris* WED57797	JQ598854	-	-	-	JQ599002
*Xenopholis undulatus* R6955	JQ598855	JQ598916	-	-	JQ599003
